# Escherichia coli-Derived Outer Membrane Vesicles Relay Inflammatory Responses to Macrophage-Derived Exosomes

**DOI:** 10.1128/mbio.03051-22

**Published:** 2023-01-17

**Authors:** Risa Imamiya, Akari Shinohara, Daisuke Yakura, Takehiro Yamaguchi, Koji Ueda, Ami Oguro, Yukiko Minamiyama, Hiroshi Ichikawa, Yasuhiko Horiguchi, Mayuko Osada-Oka

**Affiliations:** a Food Hygiene and Environmental Health, Faculty of Life and Environmental Sciences, Kyoto Prefectural University, Kyoto, Japan; b Food Hygiene and Environmental Health, Division of Applied Life Science, Graduate School of Life and Environmental Sciences, Kyoto Prefectural University, Kyoto, Japan; c Department of Medical System Protective Health and Medicine Laboratory, Graduate School of Life and Medical Sciences, Doshisha University, Kyotanabe, Japan; d Department of Pharmacology, Graduate School of Medicine, Osaka Metropolitan University, Osaka, Japan; e Project for Realization of Personalized Cancer Medicine, Cancer Precision Medicine Center, Japanese Foundation for Cancer Research, Tokyo, Japan; f Graduate School of Biomedical and Health Sciences, Hiroshima University, Hiroshima, Japan; g Department of Molecular Bacteriology, Research Institute of Microbial Diseases, Osaka University, Suita, Japan; Duke University School of Medicine

**Keywords:** extracellular vesicles, exosomes, inflammation, macrophages, outer membrane protein

## Abstract

Extracellular vesicles are considered to be an inflammatory factor in several acute and chronic inflammatory diseases. The present study shows that exosomes from macrophages (Mφ) infected with live Escherichia coli induced secretion of proinflammatory factors by uninfected Mφ. Inflammatory responses induced by exosomes derived from Mφ infected with heat-inactivated E. coli or lipopolysaccharide were significantly weaker than those elicited by outer membrane vesicles (OMVs) released from live E. coli. Proteome analysis of exosomes from Mφ infected with live or heat-inactivated E. coli revealed that E. coli proteins OmpA, GroL1, DegP, CirA, and FepA are candidate triggers of exosome-mediated inflammatory responses. OMVs from a *cirA*-deleted strain suppressed exosome-mediated inflammatory responses by uninfected Mφ. The C terminus of the CirA protein (residues 158 to 633), which was relayed from E. coli-derived OMV to Mφ-derived exosomes, promoted exosome-mediated inflammatory responses by uninfected Mφ. These results suggest an alternative mechanism by which extracellular vesicles from E. coli OMV-elicited Mφ transmit proinflammatory responses to uninfected Mφ.

## INTRODUCTION

Escherichia coli, Streptococcus pneumoniae, and Staphylococcus aureus are the most common causative microorganisms of community-acquired cases of adult sepsis (1, 2). Sepsis, one of the most serious infections of all, represents a syndrome defined as life-threatening organ dysfunction. Therefore, antimicrobial therapy is important. However, the causative organism is not identified in many cases, and blood cultures may be negative for bacteria (2). Lipopolysaccharide (LPS), an endotoxin derived from the outer membrane of Gram-negative bacteria, is one of the primary drivers of sepsis in the absence of live bacteria and has been investigated as one of the triggers of lethal shock (3). LPS triggers production of proinflammatory factors such as tumor necrosis factor alpha (TNF-α), interleukin 1 beta (IL-1β), and IL-6 by immune cells (4). These cytokines, which mediate the initial response of the innate immune system to injury or infection, are produced primarily by macrophages (Mφ). Mφ are the first line of defense against bacteria and release cellular signaling molecules and various proinflammatory cytokines and inflammatory mediators, including nitric oxide (NO) and cyclooxygenase-2 (COX-2) (5). Moreover, extracellular vesicles (EVs) released by Mφ play a clear role as inflammatory mediators. Exosomes, EVs derived from the endolysosomal pathway in mammalian cells, have a unique lipid and protein makeup (5, 6). Moreover, several cytokines, including TNF-α (7) and IL-18 (8), were reported to be contained within exosomes. Mφ-derived exosomes induce production of inflammatory factors by endothelial cells by decreasing the level of microRNA contained within exosomes (9). Therefore, exosomes are novel intercellular proinflammatory factors (10). However, the role of exosomes transmitted from infected donor Mφ to uninfected recipient Mφ is unknown. Bacteria also release EVs. EVs of Gram-negative bacteria are specifically called outer membrane vesicles (OMVs) (11). OMVs have a role in pathophysiological functions in both bacterium-bacterium and bacterium-host interactions. OMVs harbor LPS together with proteins, lipids, and virulence factors, suggesting that OMVs mediate the cytosolic localization of LPS on host cells (12). OMVs produced by Helicobacter pylori, Neisseria gonorrhoea, and Pseudomonas aeruginosa enter host epithelial cells and subsequently detect the NOD1 receptor, resulting in immune responses (13). All of the OMVs derived from Treponema denticola, Tannerella forthia, and Porphyromonas gingivalis were reported to increase TNF-α, IL-8, and IL-1β cytokine secretion (14). However, the mechanism by which bacterial OMVs elicit inflammatory responses in host cells has yet to be fully understood. In the present study, we considered the possibility that bacterial OMVs influence the ability of host cells to initiate or expand inflammation during bacterial infections, and we focused on Mφ-derived exosomes that trigger inflammatory responses in uninfected Mφ during infection with nonpathogenic E. coli or treated with E. coli-derived OMVs. Furthermore, we identified a key E. coli protein in exosomes that triggers the inflammatory responses.

## RESULTS

### Exosomes derived from live E. coli treated-Mφ promote inflammatory responses by naive Mφ.

**FIG 1:**
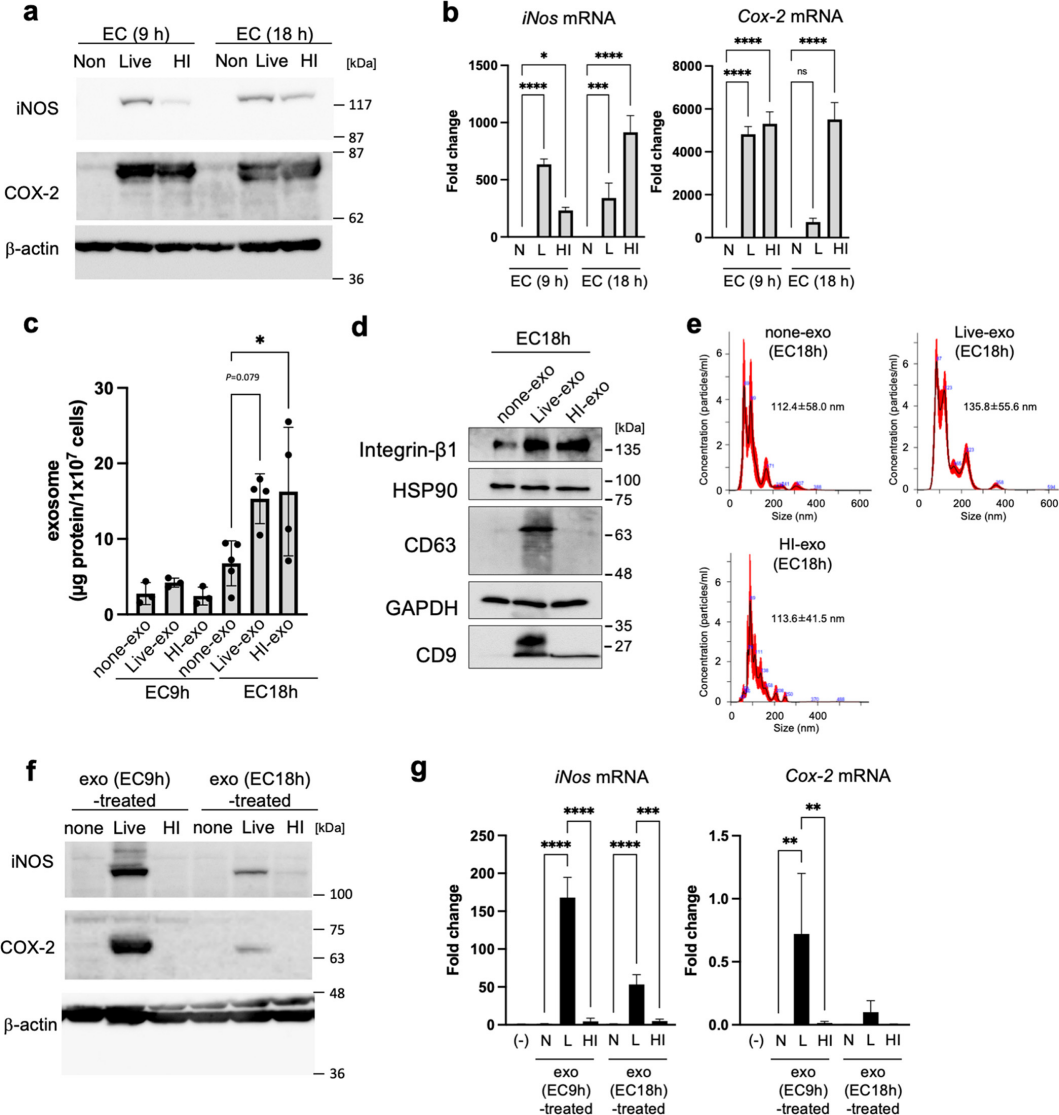
Increase in the proinflammatory mediators by exosomes from macrophages that were infected with live E. coli. After mouse RAW264.7 macrophages (Mφ) were infected for 9 or 18 h with live (L) or heat-inactivated (HI) E. coli K-12 strain (EC) at a multiplicity of infection (MOI) of 5, the exosomes (none-exo, live-exo, and HI-exo) were prepared from their culture media of cells. (a) The protein expression of inducible nitric oxide synthase (iNOS) and cyclooxygenase-2 (COX-2) in whole-cell lysates was measured by Western blotting. β-Actin was used as loading control. (b) The mRNA levels of *iNos* and *Cox-2* in bacteria-infected cells were analyzed by quantitative real-time PCR and normalized to 18S rRNA. Values are expressed as the mean ± SD (*n* = 4) of at least three independent biological replicates. (c to e) Exosomes (live-exo or HI-exo) from donor Mφ, which were infected with live or HI E. coli for 9 or 18 h, were isolated by ultracentrifugation. Exosomes (none-exo) from uninfected Mφ were collected as control. (c) The amounts of exosomes were measured by protein concentration. (d) The expression of exosome markers, including integrin β1, heat shock protein 90 (HSP90), CD63, glyceraldehyde-3-phosphate dehydrogenase (GAPDH), and CD9 in exosomes from donor Mφ, which were infected with live or HI E. coli for 18 h, were detected by Western blotting. (e) Particle size of exosomes derived from donor Mφ, which were infected with live or HI E. coli for 18 h, was analyzed by NanoSight. Values are expressed as the mean ± SD. (f, g) RAW 264.7 cells (recipient cells; 5 × 10^5^ cells) were incubated with exosomes from donor cells (1 × 10^6^ cells). (f) After incubation for 24 h, the expression of iNOS and COX-2 in whole-cell lysates was measured by Western blotting. β-Actin was used as loading control. (g) After incubation with each exosome for 24 h, the mRNA levels of *iNos* and *Cox-2* were analyzed by quantitative real-time PCR and normalized to 18S rRNA. Values are expressed as the mean ± SD (*n* = 3 to 4) of at least three independent biological replicates. One-way ANOVA and Tukey’s test were used for statistical analysis; *, *P *< 0.05; **, *P *< 0.01; ***P < 0.005; ****, *P *< 0.001; ns, not significant.

First, we examined whether exosomes derived from Mφ infected with E. coli induce expression of inflammatory mediators. Mouse RAW264.7 Mφ were infected for 9 or 18 h with live or heat-inactivated E. coli (DH5α strain) at a multiplicity of infection (MOI) of 5. Both live and heat-inactivated E. coli led to a strong increase in expression of inducible NO synthase (iNOS) and COX-2 proteins; however, expression of these proteins did not increase in noninfection (Fig. 1a). Expression of mRNA-encoding *iNos* and *Cox-2* also increased in Mφ infected with live and heat-inactivated E. coli, but not in uninfected Mφ (Fig. 1b). Next, we isolated exosomes from cell culture media by ultracentrifugation. The amount of protein in exosomes from cells infected with live or heat-inactivated E. coli (live-exo or HI-exo) for 18 h was 2.26-fold or 2.40-fold higher than that in exosomes from uninfected cells (none-exo) (Fig. 1c). Expression of integrin β1, heat shock protein 90 (HSP90), and glyceraldehyde-3-phosphate dehydrogenase (GAPDH), all of which are exosome marker proteins, was similar in all three exosomes (i.e., live-exo, HI-exo, and none-exo) (Fig. 1d). In addition, live-exo showed high expression of CD63 and CD9. Next, the particle size of each of these exosomes was analyzed using NanoSight. The diameter of live-exo (135.8 ± 55.6 nm) was a little larger than that of HI-exo (113.6 ± 41.5 nm) and none-exo (112.4 ± 58.0 nm) (Fig. 1e). When RAW264.7 Mφ were used as recipient cells, the live-exo from Mφ infected with live E. coli for 9 or 18 h showed a marked increase in expression of iNOS and COX-2 (Fig. 1f). In addition, live-exo increased expression of mRNA encoding *iNos* (9 h, 168-fold; 18 h, 53-fold) and *Cox-2* (9 h, 471-fold; 18 h, 65-fold) significantly compared with exosomes from untreated Mφ (Fig. 1g). In contrast, none-exo and HI-exo failed to increase expression of iNOS and COX-2 protein and mRNA in recipient Mφ. These data show that expression of inflammatory mediators by uninfected Mφ was increased by exposure to exosomes from donor Mφ infected with live E. coli, but not those infected by heat-inactivated E. coli.

### E. coli-derived OMVs promote exosome-mediated inflammatory responses.

**FIG 2:**
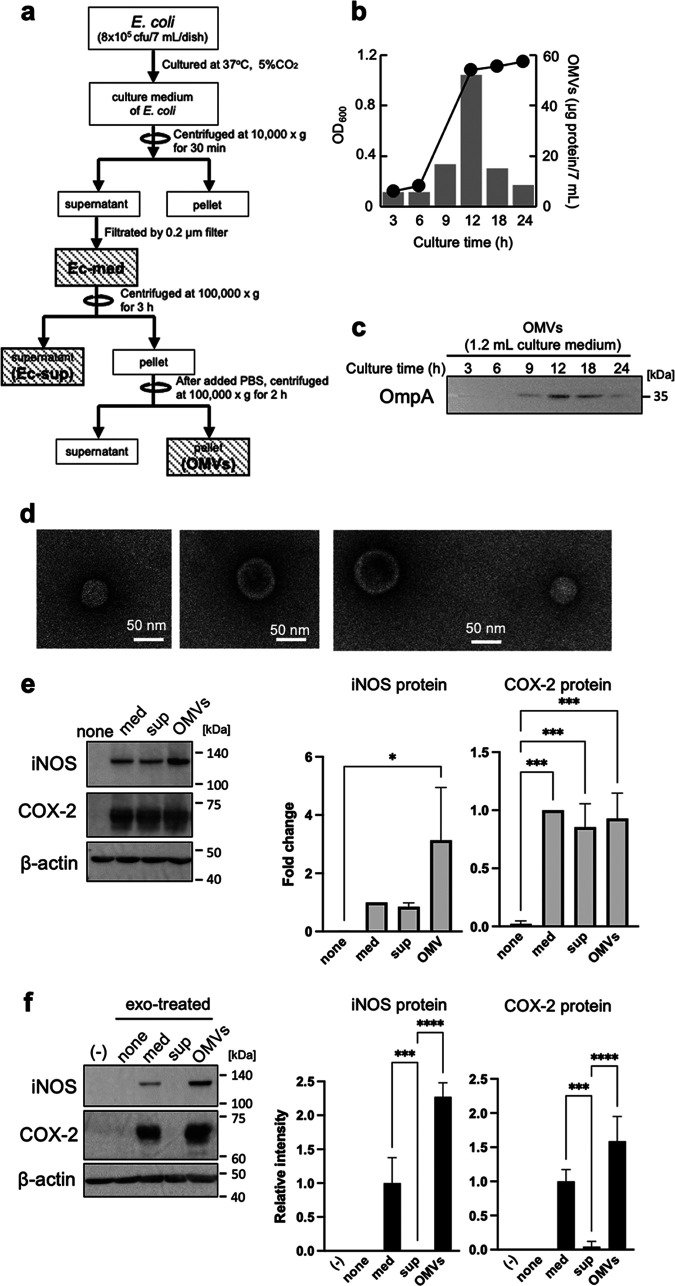
Characterization of E. coli-derived outer membrane vesicles (OMVs). (a) OMVs from the culture medium of E. coli K-12 strain during 24 h were isolated by ultracentrifugation. (b) The growth of E. coli was assessed by measuring at a wavelength of 600 nm (OD_600_) and is shown by the line graph. The number of OMV proteins is shown in the bar graph (*n* = 1). The experiment was repeated with at least three independent biological replicates. (c) OMVs from 1.2 mL of culture medium were prepared by ultracentrifugation. The protein expression of OmpA, an OMV marker, was detected by Western blotting. (d) OMVs were visualized with a transmission electron microscope. (e) After RAW264.7 cells were incubated with or without Ec-med, Ec-sup, or OMVs for 9 h, cells were incubated with fresh medium for 1 h. After we changed fresh medium again, cells were incubated for 9 h. The protein expression of inducible nitric oxide synthase (iNOS) and cyclooxygenase-2 (COX-2) in whole-cell lysates was measured by Western blotting. β-Actin was used as loading control. (f) After RAW264.7 cells (recipient cells; 5 × 10^5^ cells) were incubated with each exosome from donor cells (5 × 10^5^ cells) for 24 h, the protein expression of iNOS and COX-2 in whole-cell lysates was measured by Western blotting. β-Actin was used as loading control. The protein levels of iNOS, COX-2, and β-actin were quantitatively analyzed by using CS Analyzer 3.0 as image analysis software. Relative intensity was expressed as the mean ± SD (*n* = 3) of at least three independent biological replicates. One-way ANOVA and Tukey’s test were used for statistical analysis; *, *P *< 0.05; ***, *P *< 0.005; ****, *P *< 0.001.

Next, we focused on OMVs released by live E. coli rather than the bacteria themselves; this is because we did not prepare OMVs from heat-inactivated E. coli. To examine whether OMVs from live E. coli trigger inflammatory responses by recipient Mφ via donor Mφ-derived exosomes, we prepared OMV pellets from E. coli culture media by ultracentrifugation at 100,000 × *g* (Fig. 2a). The amount of OMVs (as protein contents) in live E. coli cultured for 3 to 24 h peaked at 12 h (Fig. 2b). Expression of OmpA, a marker of OMVs, was also highest in OMVs from E. coli cultured for 12 h (Fig. 2c). Transmission electron microscopy (TEM) showed that OMVs were about 100 nm in diameter (Fig. 2d). The amount of OMVs and OmpA decreased in a time-dependent manner after 12 h; therefore, we prepared OMVs from E. coli cultured for 12 h. After incubation of donor Mφ with E. coli culture medium (med) for 9 h, the supernatant (sup), or the pellet (OMVs) (the latter two were obtained from E. coli culture media by ultracentrifugation at 100,000 × *g*), the Mφ medium was exchanged with fresh medium. Exosomes were then collected again from Mφ culture medium after 9 h. We found that expression of iNOS and COX-2 proteins by donor Mφ treated with med, sup, or the OMV pellet was higher than that by untreated Mφ (Fig. 2e). In recipient Mφ, expression of iNOS and COX-2 increased markedly after exposure to exosomes from Mφ treated with the med (relative intensity of iNOS, 1.0, and COX-2, 1.0) and OMVs (iNOS, 2.27; COX-2, 1.59), but not by exosomes from Mφ treated with sup (iNOS, 0; COX-2, 0.04) (Fig. 2f). These results suggest that OMVs derived from live E. coli increase the exosome-mediated inflammatory responses by uninfected Mφ. Next, we confirmed the relationship between OMV concentration and exosome-mediated inflammatory responses. In donor Mφ, the levels of iNOS and COX-2 proteins in Mφ (8 × 10^6^ cells) treated with a low concentration of OMVs (0.5 μg/7 mL) were the same as in Mφ (8 × 10^6^ cells) treated with a large concentration of OMVs (25 μg/7 mL) (see Fig. S1a in the supplemental material). In contrast, exosomes from Mφ treated with a high concentration of OMVs (5, 10, or 25 μg/7 mL) showed increased levels of iNOS and COX-2 proteins, while exosomes from Mφ treated with a low concentration of OMVs (0.5 or 1.0 μg/7 mL) did not (Fig. S1b). These results indicate that there is a threshold concentration of OMVs that triggers exosome-mediated inflammatory responses. Next, we examined whether LPS promoted exosome-mediated inflammatory responses; this is because OMVs derived from the outer membrane of E. coli contain a large amount of LPS (12). LPS increased expression of iNOS and COX-2 proteins by donor Mφ, whereas exosomes from Mφ treated with LPS did not increase protein expression by naive Mφ (Fig. S2a and b). LPS increased expression of iNOS and COX-2 in a dose-dependent manner; the data suggest that LPS at a dose of >1 ng/mL in culture medium is required to increase expression of inflammatory mediators (Fig. S2c). In contrast, when we measured the concentration of LPS in exosomes from Mφ treated with live E. coli, it was 0.0025 ± 0.0001 ng/mL of culture medium (Fig. S2d). We examined the levels of expression of inflammatory mediators by using ΔLPS-OMVs derived from E. coli (ClearColi), which deleted the two secondary acyl chains of the normally hexa-acylated LPS, in recipient cells. ΔLPS-OMVs increased the expression of iNOS and COX-2 proteins by donor Mφ (Fig. S2f). Exosomes from Mφ treated with ΔLPS-OMVs also increased the mRNA levels of inflammatory mediators by naive Mφ. These results suggest that exosome-mediated inflammatory responses are triggered by molecules other than LPS.

10.1128/mbio.03051-22.1 FIG S1 Exosomes caused inflammatory responses in OMVs dose dependently. Mouse RAW264.7 macrophages (Mφ) were incubated with OMVs (0 to 25 μg/7 mL) derived from E. coli K-12 culture medium. RAW264.7 cells were incubated with OMVs at the indicated concentration for 9 h. Culture media were replaced with media containing antibiotics, and Mφ were cultured for 1 h. After we changed the new media again, Mφ were cultured for 9 h. Exosomes isolated from culture medium of donor Mφ. RAW 264.7 cells were incubated with exosomes (0.7 μg/mL) for 24 h. The expression of inducible nitric oxide synthase (iNOS) and cyclooxygenase-2 (COX-2) in cell lysates of donor Mφ (a) and recipient Mφ (b) was measured by Western blotting. β-Actin was used as loading control. CS Analyzer 3.0 was used as image analysis software. Data are the mean ± SD (*n* = 3). ANOVA and Mann-Whitney U test were used for statistical analysis. *, *P* < 0.05; ***, *P* < 0.005; ****, *P* < 0.001. Download FIG S1, PDF file, 2.4 MB. Copyright © 2023 Imamiya et al. 2023 Imamiya et al. https://creativecommons.org/licenses/by/4.0/ This content is distributed under the terms of the Creative Commons Attribution 4.0 International license.

10.1128/mbio.03051-22.2 FIG S2 LPS was not associated with inflammatory responses by macrophage-derived exosomes. (a) Mouse RAW264.7 macrophages (Mφ) were incubated with 100 ng/mL lipopolysaccharide (LPS) or a heat-inactivated (HI) E. coli K-12 strain (MOI of 5) for 9 h. Culture medium was replaced with medium containing antibiotics, and Mφ were cultured for 1 h. After we changed the new medium again, Mφ were cultured for 9 h. Exosomes were isolated from the culture medium of donor Mφ. (b) RAW 264.7 cells were incubated with exosomes (0.7 μg/mL) for 24 h. (c) RAW264.7 cells were incubated with 0 to 10 ng/mL LPS or live-exo (0.7 μg/mL). The protein expression of iNOS and COX-2 in cell lysates of Mφ was measured by Western blotting. β-Actin was used as loading control. (d) LPS concentration in culture medium of recipient Mφ, which were treated with 0.7 μg/mL live-exo, were analyzed by using *Limulus* amebocyte lysate (LAL). (e) ΔLPS-OMVs from 0.5 mL of culture medium of ClearColi were prepared by ultracentrifugation. The protein expression of OmpA and CirA was detected by Western blotting. (f) RAW264.7 cells were incubated with or without ΔLPS OMVs (10 μg/7 mL) for 9 h. After the culture medium was replaced with fresh medium containing antibiotics, cells were incubated for 1 h. After we changed the fresh medium again, cells were incubated for 9 h. The protein expression of iNOS and COX-2 in whole-cell lysates was measured by Western blotting. β-Actin was used as loading control. (g) RAW264.7 cells were incubated with exosomes (0.7 μg/mL) for 12 h. The mRNA expression of iNos, Cox-2, tumor necrosis factor alpha (TNF-α), interleukin 1β (IL-1β), and IL-6 was analyzed by quantitative real-time PCR and normalized to 18S rRNA. Data are the mean ± SD (*n* = 3). A *t* test was used for statistical analysis. *, *P* < 0.05; **, *P* < 0.01. Download FIG S2, PDF file, 2.5 MB. Copyright © 2023 Imamiya et al. 2023 Imamiya et al. https://creativecommons.org/licenses/by/4.0/ This content is distributed under the terms of the Creative Commons Attribution 4.0 International license.

Furthermore, we examined whether exosomes from mouse bone marrow-derived macrophages (BMDMs) treated with E. coli-derived OMVs enhanced inflammatory responses. E. coli-derived OMVs increased the expression of iNOS and COX-2 proteins by donor Mφ of BMDM (Fig. S3a). Mouse BMDM-derived exosomes increased the mRNA levels of inflammatory mediators by naive BMDM (Fig. S3c).

10.1128/mbio.03051-22.3 FIG S3 Exosomes from mouse BMDMs treated by OMVs upregulated inflammatory mediator. (a) DMDMs were incubated with or without OMVs (10 μg/7 mL) for 9 h. After the culture medium was replaced with fresh medium containing antibiotics, cells were incubated for 1 h. After we changed fresh medium again, cells were incubated for 9 h. The protein expression of inducible nitric oxide synthase (iNOS) and cyclooxygenase-2 (COX-2) in whole-cell lysates was measured by Western blotting. β-Actin was used as loading control. (b) Exosomes isolated from culture medium of donor Mφ. The expression of exosome markers inducing heat shock protein 90 (HSP90) and glyceraldehyde-3-phosphate dehydrogenase (GAPDH) in exosomes were detected by Western blotting. (c) Mouse BMDMs were incubated with exosomes (0.7 μg/mL) for 12 h. The mRNA expression of *iNos*, *Cox-2*, tumor necrosis factor alpha (TNF-α), interleukin 1β (IL-1β), and IL-6 were analyzed by quantitative real-time PCR and normalized to 18S rRNA. Data are the mean ± SD (*n* = 3). A *t* test was used for statistical analysis. *, *P* < 0.05. Download FIG S3, PDF file, 1.9 MB. Copyright © 2023 Imamiya et al. 2023 Imamiya et al. https://creativecommons.org/licenses/by/4.0/ This content is distributed under the terms of the Creative Commons Attribution 4.0 International license.

### Proteins contained within exosomes promote inflammatory responses.

**FIG 3:**
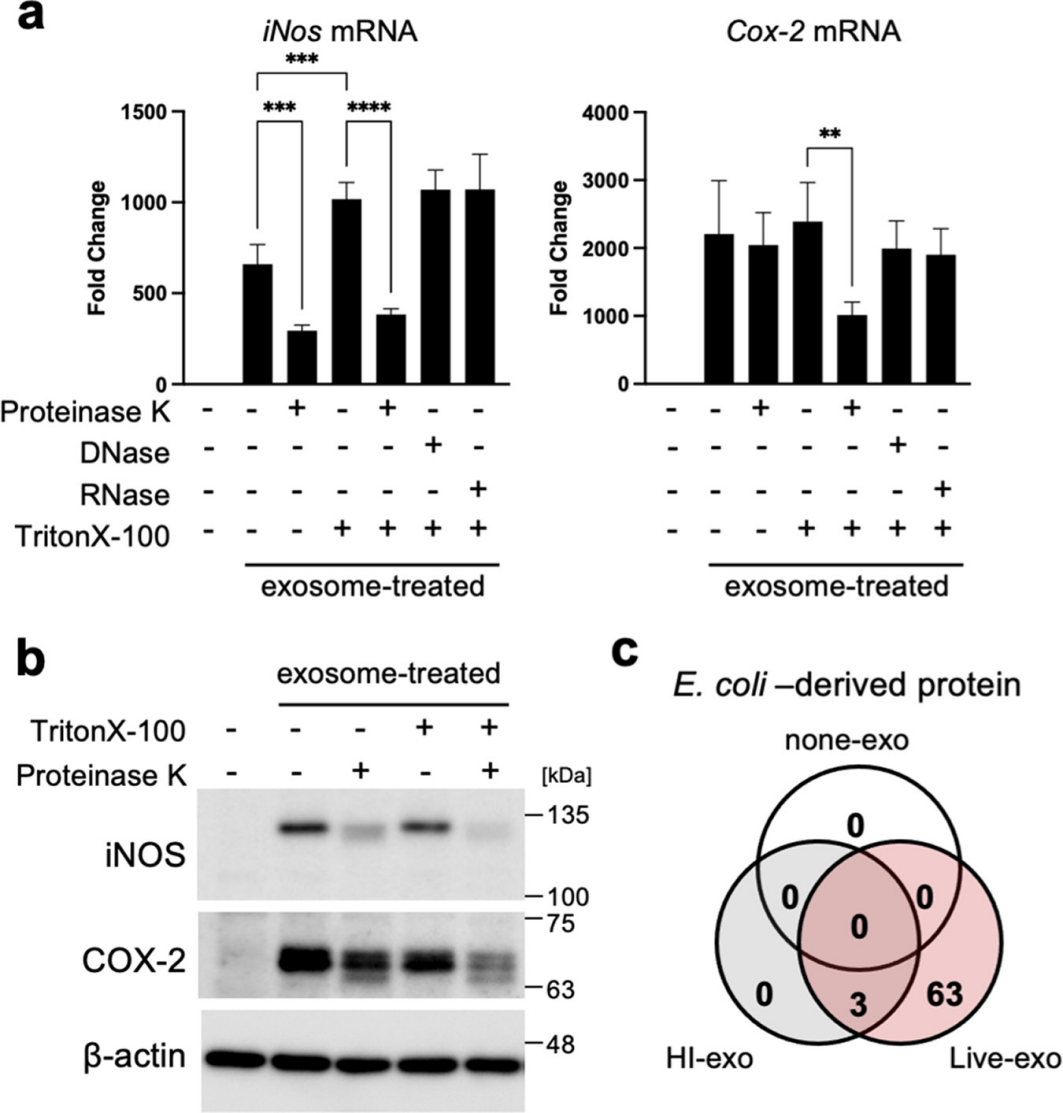
E. coli proteins in exosomes were candidates as important factors in inflammatory responses. (a, b) Exosomes from live E. coli-infected macrophages (Mφ) were treated with proteinase K (150 μg/mL), DNase (100 μg/mL), or RNase (100 μg/mL) in the presence or absence of 0.01% Triton X-100. Mouse RAW264.7 Mφ was incubated with each exosome (0.1 μg/mL). (a) After incubation of cells for 24 h, the mRNA levels of inducible nitric oxide synthase (*iNos*) and cyclooxygenase-2 (*Cox-2*) were analyzed by quantitative real-time PCR and normalized to 18S rRNA. Values are expressed as the mean ± SD (*n* = 4) of at least three independent biological replicates. One-way ANOVA and Tukey’s test were used for statistical analysis; **, *P *< 0.01; ***, *P *< 0.005; ****, *P *< 0.001. (b) After incubation of cells for 24 h, the protein expressions of iNOS and COX-2 were measured by Western blotting. β-Actin was used as loading control. (c) Exosomes (none-exo, live-exo, or HI-exo) from Mφ uninfected or infected with live or heat-inactive (HI) E. coli were analyzed by mass spectrometry. Venn diagrams show the number of identified proteins.

To examine the components within exosomes that increase expression of inflammatory mediators, exosomes from Mφ infected with live E. coli were treated with proteinase K, DNase, or RNase in the presence or absence of Triton X-100. Live-exo treated with proteinase K in the presence or absence of Triton X-100 failed to increase expression of iNOS protein and mRNA significantly (Fig. 3a and b). However, exosome-mediated increases in expression of COX-2 protein and mRNA were inhibited by proteinase K in the presence of Triton X-100. In contrast, DNase and RNase did not suppress the exosome-mediated increase in expression of *iNos* and *Cox-2* mRNA (Fig. 3a). These results show that the proteins contained within exosomes are an important factor that triggers exosome-mediated inflammatory responses. Therefore, we used MS analysis to examine proteins contained in three exosomes (none-exo, live-exo, and HI-exo) from Mφ infected with or without live or heat-inactivated E. coli. E. coli proteins and/or mouse Mφ proteins were detected in all three exosomes. Data shown earlier demonstrated that increased exosome-mediated inflammatory responses in recipient Mφ were dependent on the concentration of OMVs in donor Mφ (Fig. S1); therefore, we focused on proteins expressed by E. coli. Sixty-six E. coli proteins were detected from live-exo. Among these, only three proteins were also found in HI-exo (Fig. 3c; Table 1). OmpA, GroL1, DegP, CirA, and FepA proteins were identified as candidate triggers of exosome-mediated inflammatory responses because large numbers of their peptide fragments were detected by mass spectrometry (MS). Aldehyde-alcohol dehydrogenase (ADHE) was the most abundant peptide fragment detected in all E. coli proteins from live-exo; however, ADHE was removed as a candidate because mouse ADHE was contained in all three exosomes from Mφ.

**TABLE 1:** Exclusive unique peptide content^*a*^

No.	Identified protein	Accession no.	Live_1	Live_2	HI_1	HI_2	None_1	None_2	OMVs
1	Aldehyde-alcohol dehydrogenase (adhE)	ADHE_ECO57	58	55	0	0	0	0	+
2	Outer membrane protein A (ompA)	OMPA_ECO57	32	45	3	2	0	0	+
3	60-kDa chaperonin 1 (groL1)	CH601_ECOK1	28	30	0	0	0	0	+
4	Periplasmic serine endoprotease DegP (degP)	DEGP_ECO57	22	14	0	0	0	0	+
5	Fe^3+^ dicitrate transport protein FecA (fecA)	FECA_ECOLI	20	13	0	0	0	0	+
6	Colicin I receptor (car)	CIRA_ECOLI	20	26	0	1	0	0	+
7	Outer membrane protein TolC (tolC)	TOLC_ECOLI	20	17	0	1	0	0	+
8	Uncharacterized protein YncE (yncE)	YNCE_ECOLI	19	16	0	0	0	0	+
9	Ferrienterobactin receptor (fepA)	FEPA_ECOLI	18	19	0	0	0	0	+
10	Vitamin B_12_ transporter BtuB (btuB)	BTUB_ECOLI	17	8	0	0	0	0	+
11	30S ribosomal protein S4 (rpsD)	RS4_ECO24	17	17	0	0	0	0	+
12	Dihydrolipoyllysine-residue acetyltransferase component of pyruvate dehydrogenase complex (aceF)	ODP2_ECOLI	15	11	0	0	0	0	+
13	30S ribosomal protein S7 (rpsG)	RS7_ECOBW	15	13	0	0	0	0	+
14	DNA-directed RNA polymerase subunit beta′ (rpoC)	RPOC_ECO24	14	16	1	0	0	0	+
15	Outer membrane protease OmpP (ompP)	OMPP_ECOLI	14	6	0	0	0	0	+
16	Protein TolB (tolB)	TOLB_ECO24	13	9	0	0	0	0	+
17	Elongation factor Tu 1 (tuf1)	EFTU1_ECO24	13	11	0	0	0	0	+
18	Outer membrane protein X (ompX)	OMPX_ECO57	13	9	0	1	0	0	+
19	Metal-binding protein ZinT (zinT)	ZINT_ECOLI	12	9	0	0	0	0	
20	Major outer membrane lipoprotein (lpp)	LPP_ECO57	12	15	1	0	0	0	+
21	Cell division coordinator CpoB (cpoB)	CPOB_ECOLI	11	6	0	0	0	0	
22	High-affinity zinc uptake system protein ZnuA (znuA)	ZNUA_ECOLI	10	10	0	0	0	0	+
23	Type 1 fimbrial protein, A chain (fimA)	FIMA1_ECOLI	10	12	3	4	0	0	
24	Outer membrane protein assembly factor BamA (bamA)	BAMA_ECO24	8	9	0	0	0	0	
25	Glyceraldehyde-3-phosphate dehydrogenase A (gapA)	G3P1_ECO57	8	4	0	0	0	0	
26	30S ribosomal protein S13 (rpsM)	RS13_ECO57	8	9	0	0	0	0	+
27	Ribose import binding protein RbsB (rbsB)	RBSB_ECOLI	8	6	0	0	0	0	+
28	50S ribosomal protein L17 (rplQ)	RL17_ECO24	7	8	0	0	0	0	+
29	FKBP-type peptidyl-prolyl *cis*-*trans* isomerase FkpA (fkpA)	FKBA_ECO57	7	8	0	0	0	0	
30	Putative acyl-CoA thioester hydrolase YbhC (ybhC)	YBHC_ECOLI	7	5	0	0	0	0	+
31	Chaperone protein Skp (skp)	SKP_ECO57	7	3	0	0	0	0	+
32	Outer membrane lipoprotein SlyB (slyB)	SLYB_ECO57	7	4	0	0	0	0	+
33	50S ribosomal protein L21 (rplU)	RL21_ECO24	7	3	0	0	0	0	
34	DNA-directed RNA polymerase subunit alpha (rpoA)	RPOA_ECO24	6	11	0	1	0	0	
35	Peptidoglycan-associated lipoprotein (pal)	PAL_ECO57	6	7	0	0	0	0	+
36	Phosphate-binding protein PstS (pstS)	PSTS_ECOLI	6	3	0	0	0	0	
37	50S ribosomal protein L4 (rplD)	RL4_ECO24	6	8	0	0	0	0	
38	Outer membrane protein assembly factor BamC (bamC)	BAMC_ECOLI	6	5	0	0	0	0	
39	Uncharacterized protein YegR (yegR)	YEGR_ECOLI	6	2	0	0	0	0	+
40	Protein FimH (fimH)	FIMH_ECOLI	6	4	0	0	0	0	
41	50S ribosomal protein L20 (rplT)	RL20_ECO24	5	10	0	0	0	0	+
42	Uncharacterized protein YqjD (yqjD)	YQJD_ECO57	5	3	0	0	0	0	+
43	Outer membrane protein assembly factor BamB (bamB)	BAMB_ECOLI	5	6	0	0	0	0	
44	50S ribosomal protein L24 (rplX)	RL24_ECO24	5	3	0	0	0	0	
45	50S ribosomal protein L2 (rplB)	RL2_ECO24	4	6	0	0	0	0	+
46	50S ribosomal protein L1 (rplA)	RL1_ECO27	4	7	0	0	0	0	+
47	Uncharacterized lipoprotein YajG (yajG)	YAJG_ECOL6	4	8	0	0	0	0	+
48	Penicillin-binding protein activator LpoA (lpoA)	LPOA_ECOLI	4	5	0	3	0	0	
49	Rare lipoprotein A (rlpA)	RLPA_ECOLI	4	3	0	0	0	0	+
50	Protein YdgH (ydgH)	YDGH_ECOLI	4	2	0	0	0	0	+
51	50S ribosomal protein L15 (rplO)	RL15_ECO24	4	4	0	0	0	0	
52	30S ribosomal protein S6 (rpsF)	RS6_ECO24	4	7	0	0	0	0	
53	Outer membrane protein (ompC)	OMPC_ECOLI	3	7	0	0	0	0	+
54	Protein TraN GN (traN)	TRAN_ECOLI	3	2	0	0	0	0	
55	Chaperone protein FimC (fimC)	FIMC_ECOL6	3	2	0	0	0	0	
56	30S ribosomal protein S9 (rpsI)	RS9_ECO24	3	6	0	0	0	0	+
57	LPS assembly protein LptD (lptD)	LPTD_ECOK1	3	3	0	0	0	0	
58	TraT complement resistance protein (traT)	TRAT1_ECOLI	3	6	0	0	0	0	
59	Nucleoside-specific channel-forming protein tsx (tsx)	TSX_ECO57	3	4	0	0	0	0	+
60	Ferrichrome-iron receptor (fhuA)	FHUA_ECOLI	3	3	0	0	0	0	+
61	30S ribosomal protein S2 (rpsB)	RS2_ECO27	2	5	0	0	0	0	+
62	Uncharacterized protein YggE (yggE)	YGGE_ECO57	2	1	0	0	0	0	
63	Inhibitor of g-type lysozyme (pliG)	PLIG_ECOLI	2	3	0	0	0	0	
64	50S ribosomal protein L13 (rplM)	RL13_ECO24	2	1	0	0	0	0	+
65	DNA-directed RNA polymerase subunit beta (rpoB)	RPOB_ECOL5	1	4	0	0	0	0	+
66	50S ribosomal protein L5 (rplE)	RL5_ECO24	1	4	0	0	0	0	+

a Live-exo (Live), HI-exo (HI), and none-exo (None) (n = 2) were analyzed by mass spectrometry. The number represent total spectrum count. The proteins reported to be present in OMVs (ref. 15) were indicated by “+” in TABLE 1.

### Single gene-deleted E. coli suppresses exosome-mediated inflammatory responses.

**FIG 4:**
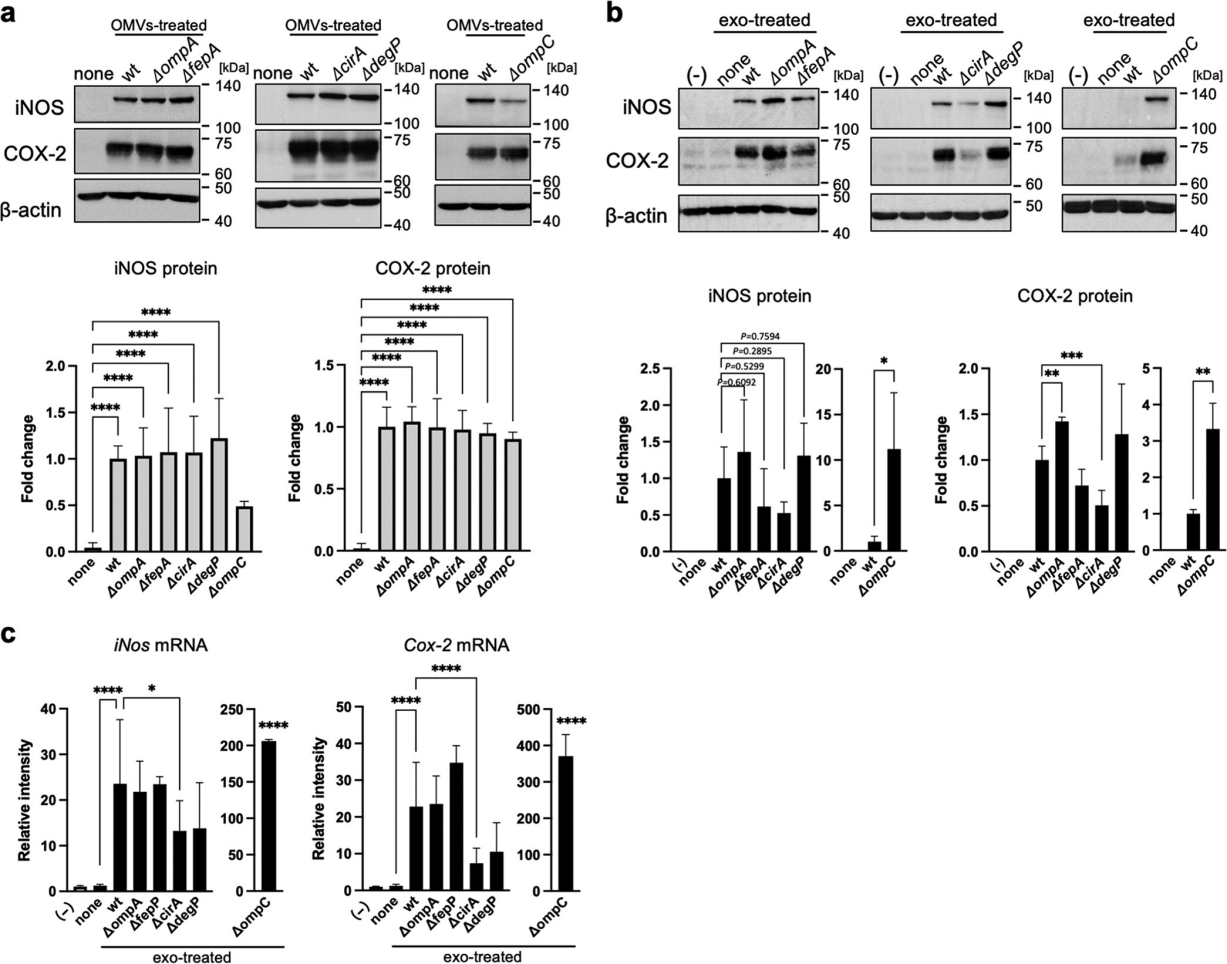
E. coli cirA-deleted mutant strain suppressed the exosome-mediated inflammatory responses. (a) Mouse RAW264.7 macrophages (Mφ) were incubated with OMVs (10 μg/7 mL) derived from single gene-deleted mutant strains (*ΔompA*, *ΔompC*, *ΔfepA*, *ΔcirA*, or *ΔdegP*) and wild type (WT) for 9 h. After culture media were replaced with fresh media containing antibiotics, cells were incubated for 1 h. After replacement with fresh media containing antibiotics, cells were incubated for 9 h. The protein expression of inducible nitric oxide synthase (iNOS) and cyclooxygenase-2 (COX-2) in whole-cell lysates was measured by Western blotting. β-Actin was used as loading control. (b) RAW264.7 cells (recipient cells; 5 × 10^5^ cells) were incubated with each exosome from donor cells (5 × 10^5^ cells) for 24 h. The protein expression of iNOS and COX-2 in whole-cell lysates was measured by Western blotting. β-Actin was used as loading control. CS Analyzer 3.0 was used for image analysis software. Relative intensity is expressed as the mean ± SD (*n* = 3) of at least three independent biological replicates. (c) RAW264.7 cells (recipient cells; 5 × 10^5^ cells) were incubated with each exosome from donor cells (5 × 10^5^ cells) for 24 h. The mRNA expression of *iNos* and *Cox-2* was analyzed by quantitative real-time PCR and normalized to 18S rRNA. Values are expressed as the mean ± SD (*n* = 4) of at least three independent biological replicates. One-way ANOVA and Tukey’s test were used for statistical analysis; *, *P *< 0.05; **, *P *< 0.01; ***, *P *< 0.005; ****, *P *< 0.001; ns, not significant.

To determine which factors are important for triggering exosome-mediated inflammatory responses, we next examined whether OMVs from single gene-deleted E. coli strains (i.e., *ΔompA*, *ΔfepA*, *ΔcirA*, and *ΔdegP*) obtained from the Keio library triggered exosome-mediated inflammatory responses. Since *groE1* is a lethal gene, we did not examine it in this study. Moreover, strain *ΔompC* was used as a negative control because OmpC is an abundant protein in OMVs. Density gradient ultracentrifugation was used to confirm expression of the OmpA, FepA, CirA, DegP, and OmpC proteins in OMVs. All proteins were most abundant in fraction 9 (Fig. S4a). The density on fraction 9 (1.16 g/mL) was lower than that reported for OMVs (1.2 g/mL) (15, 16) (Fig. S4b). Next, OMVs from each single gene-deleted E. coli strain were prepared by centrifugation. OMVs derived from the *ΔompA*, *ΔfepA*, *ΔcirA*, *ΔdegP*, and *ΔompC* strains contained five proteins (Fig. S4c). The increased expression of iNOS and COX-2 proteins by donor Mφ exposed to OMVs was the same for the wild-type (WT) strain and the five single gene-deleted strains (Fig. 4a). In recipient Mφ, expression of iNOS and COX-2 proteins increased in the presence of exosomes from Mφ treated with OMVs from the *ΔompA*, *ΔompC*, *ΔfepA*, and *ΔdegP* strains ((*ΔompA*-exo, *ΔompC*-exo, Δ*fepA*-exo, and Δ*degP*-exo, respectively) (Fig. 4b)). In contrast, expression of COX-2 protein by exosomes from Mφ-treated OMVs from the *ΔcirA* strain (*ΔcirA*-exo) decreased 0.51-fold in comparison with treatment by OMVs from the WT strain (WT-exo). Expression of iNOS by *ΔcirA*-exo decreased 0.52-fold compared with that by OMVs from the WT strain, albeit not significantly. Similar to protein levels, the increase in expression of *iNos* and *Cox-2* mRNA decreased 0.56-fold and 0.32-fold, respectively, in recipient Mφ exposed to *ΔcirA*-exo compared to WT-exo (Fig. 4c). The increase in iNOS and COX-2 protein and mRNA in recipient Mφ treated with OMVs from the Δ*ompC* strain was significantly higher than after treatment with OMVs from the WT strain (iNOS protein, 11.2-fold; *iNos* mRNA, 206-fold; COX-2 protein, 3.3-fold; and *cox-2* mRNA, 370-fold). In addition, expression of TNF-α and IL-1β was decreased by *ΔcirA*-exo to a greater extent than by WT-exo (Fig. S5). These results suggest that the *cirA*-deleted strain suppressed exosome-mediated inflammatory responses.

10.1128/mbio.03051-22.4 FIG S4 Density of each fraction after centrifugation of density gradient. (a) OMVs were isolated from the culture medium of E. coli K-12 strain by density gradient ultracentrifugation. Gradient fractions were collected and analyzed by Western blotting with anti-OmpA, anti-FepA, anti-CirA, anti-DegP, and anti-OmpC antibodies. (b) After OMVs were separated on a 5 to 40% iodixanol gradient, the densities of 12 fractions were calculated by measuring the absorbance at a wavelength of 244 nm. (c) OMVs from a single gene-deleted strain (*ΔompA*, *ΔompC*, *ΔfepA*, *ΔcirA*, or *ΔdegP*) were analyzed by Western blotting. (d) The number of OMVs is shown. Data are the mean ± SD. Download FIG S4, TIF file, 0.7 MB. Copyright © 2023 Imamiya et al. 2023 Imamiya et al. https://creativecommons.org/licenses/by/4.0/ This content is distributed under the terms of the Creative Commons Attribution 4.0 International license.

10.1128/mbio.03051-22.5 FIG S5 *cirA*-deleted E. coli suppressed the mRNA levels of proinflammatory factors by macrophage-derived exosomes. Mouse RAW264.7 cells (recipient cells; 5 × 10^5^ cells) were incubated with exosomes from donor cells (5 × 10^5^ cells) for 24 h. The mRNA expression levels of tumor necrosis factor alpha (TNF-α) (a), interleukin 1 beta (IL-1β) (b), and IL-6 (c) were analyzed by quantitative real-time PCR and normalized to 18S rRNA. Values are expressed as the mean ± SD (*n* = 4) of at least three independent biological replicates. One-way ANOVA and Tukey’s test were used for statistical analysis; *, *P* < 0.05; **, *P* < 0.01; ***, *P* < 0.005; ****, *P* < 0.001. Download FIG S5, PDF file, 0.2 MB. Copyright © 2023 Imamiya et al. 2023 Imamiya et al. https://creativecommons.org/licenses/by/4.0/ This content is distributed under the terms of the Creative Commons Attribution 4.0 International license.

### Complementation of the *cirA* gene promotes exosome-mediated inflammatory responses.

**FIG 5:**
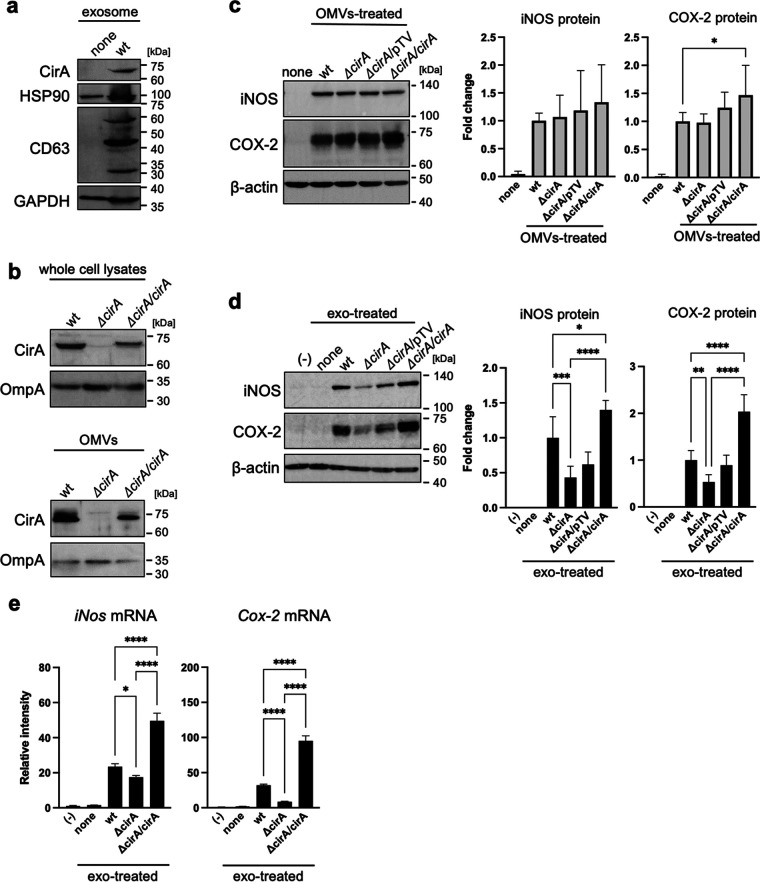
Complementation of the *cirA* gene rescued the exosome-mediated inflammatory responses. (a) The protein expression of exosome markers, including CirA, heat shock protein 90 (HSP90), CD63, and glyceraldehyde-3-phosphate dehydrogenase (GAPDH) in exosomes, which were isolated from cell culture medium by ultracentrifugation, was measured by Western blotting. (b) OMVs were isolated from culture medium of E. coli wild type (WT), *cirA* gene-deleted mutant strains (*ΔcirA*), the *ΔcirA* strain transformed with pTV-118N vector or pTV-*cirA* vector (*ΔcirA*/pTV or *ΔcirA*/*cirA*) by ultracentrifugation. The protein expression of CirA in whole-cell lysates and OMVs was measured by Western blotting. OmpA was used as loading control. (c) Mouse RAW264.7 macrophages (Mφ) were incubated with OMVs (10 μg/7 mL) for 9 h. After the culture medium was replaced with fresh medium containing antibiotics, cells were incubated for 1 h. After replacement with fresh media containing antibiotics again, cells were incubated for 9 h. The protein expression of inducible nitric oxide synthase (iNOS) and cyclooxygenase-2 (COX-2) in whole-cell lysates was measured by Western blotting. β-Actin was used as loading control. CS Analyzer 3.0 was used for image analysis software. Relative intensity was expressed as the mean ± SD (*n* = 3) of at least three independent biological replicates. (d) RAW264.7 cells (recipient cells; 5 × 10^5^ cells) were incubated with exosomes from donor cells (5 × 10^5^ cells). After incubation of cells for 24 h, the protein expression of iNOS and COX-2 in whole-cell lysates was measured by Western blotting. β-Actin was used as loading control. CS Analyzer 3.0 was used for image analysis software. Relative intensity is expressed as the mean ± SD (*n* = 3) of at least three independent experiments. (e) RAW264.7 cells (recipient cells; 5 × 10^5^ cells) were incubated with exosomes from donor cells (5 × 10^5^ cells). After incubation of cells for 12 h, the mRNA levels of *iNos* and *Cox-2* were analyzed by quantitative real-time PCR and normalized to 18S rRNA. Values are expressed as the mean ± SD (*n* = 4) of at least three independent biological replicates. One-way ANOVA and Tukey’s test were used for statistical analysis; *, *P *< 0.05; **, *P *< 0.01; ***, *P *< 0.005; ****, *P *< 0.001.

Next, we examined whether WT-exo contained the CirA protein. WT-exo, but not none-exo, contained the CirA protein together with exosome marker proteins HSP90, CD63, and GAPDH (Fig. 5a). To confirm that CirA is a key factor in exosome-mediated inflammatory responses, a pTV-cirA vector expressing CirA protein, or a pTV-118N empty vector was transformed into the E. coli *ΔcirA* strain. In bacterial cells and OMVs, the protein levels of CirA in the *cirA* gene-complementary strain (*ΔcirA/cirA*) were the same as those in the WT strain (Fig. 5b). The increase in iNOS and COX-2 proteins in donor Mφ treated by OMVs was the same after exposure to the WT, *ΔcirA* strain, and *ΔcirA*/pTV and *ΔcirA/cirA* strains (Fig. 5c). However, in recipient Mφ, the increase in iNOS and COX-2 mediated by *ΔcirA/cirA*-exo was the same as the mediated by WT-exo, while that mediated by Δ*cirA*-exo and *ΔcirA*/pTV-exo was lower than that mediated by WT-exo (Fig. 5d). The increase in the mRNA levels of *iNos*, *Cox-2*, TNF-α, IL-1β, and IL-6 mediated by *ΔcirA/cirA*-exo and WT-exo was higher than that mediated by *ΔcirA*-exo (Fig. 5e; Fig. S6c). Therefore, CirA plays an important role in exosome-mediated inflammatory responses.

10.1128/mbio.03051-22.6 FIG S6 Complementation of the 5*cirA* gene increased the expression of cytokines mRNA by macrophage-derived exosomes. (a) The number of OMVs is shown. (b) RAW264.7 cells (recipient cells; 5 × 10^5^ cells) were incubated with exosomes from donor cells (5 × 10^5^ cells) for 24 h. The mRNA expression levels of tumor necrosis factor alpha (TNF-α), interleukin 1 beta (IL-1β), and IL-6 were analyzed by quantitative real-time PCR and normalized to 18S rRNA. Values are expressed as the mean ± SD (*n* = 4) of at least three independent biological replicates. One-way ANOVA and Tukey’s test were used for statistical analysis. *, *P* < 0.05; **, *P* < 0.01; ***, *P* < 0.005; ****, *P* < 0.001. Download FIG S6, PDF file, 0.1 MB. Copyright © 2023 Imamiya et al. 2023 Imamiya et al. https://creativecommons.org/licenses/by/4.0/ This content is distributed under the terms of the Creative Commons Attribution 4.0 International license.

### The C-terminal protein of CirA promotes exosome-mediated inflammatory responses.

**FIG 6:**
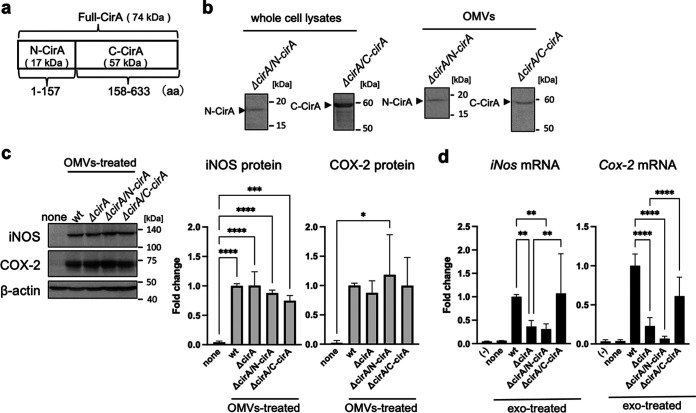
C-terminal CirA caused exosome-mediated inflammatory responses. (a) The molecular weight and structure of N-terminal and C-terminal CirA protein (N-CirA and C-CirA) are shown. (b to d) OMVs were isolated from the culture medium of E. coli wild type (WT), *cirA* gene-deleted mutant strains (*ΔcirA*), and *ΔcirA* transformed with pTV-N-cirA vector or pTV-C-cirA vector (*ΔcirA*/N-*cirA* or *ΔcirA*/C-*cirA*) by ultracentrifugation. (b) The protein expression of N-CirA and C-CirA in whole-cell lysates and OMVs was measured by Western blotting. (c) Mouse RAW264.7 macrophages (Mφ) were incubated with OMVs (10 μg/7 mL) for 9 h. After the culture medium was replaced with fresh medium containing antibiotics, cells were incubated for 1 h. After replacement with fresh media containing antibiotics again, cells were incubated for 9 h. The protein expression of inducible nitric oxide synthase (iNOS) and cyclooxygenase-2 (COX-2) in whole-cell lysates was measured by Western blotting. β-Actin was used as loading control. CS Analyzer 3.0 was used for image analysis software. Relative intensity was expressed as the mean ± SD (*n* = 3) of at least three independent biological replicates. (d) Exosomes were isolated from cell culture medium by ultracentrifugation. RAW264.7 cells (recipient cells; 5 × 10^5^ cells) were incubated with each exosome from donor cells (5 × 10^5^ cells). After incubation for 12 h, the mRNA levels of *iNos* and *Cox-2* were analyzed by quantitative real-time PCR and normalized to 18S rRNA. Values are expressed as the mean ± SD (*n* = 6 to 9) of at least three independent biological replicates. One-way ANOVA and Tukey’s test were used for statistical analysis; *, *P *< 0.05; **, *P *< 0.01; ***, *P *< 0.005; ****, *P *< 0.001.

To identify the CirA region responsible for the increase in protein and mRNA expression of proinflammatory mediators in recipient Mφ, we divided the CirA protein into two fragments, an N-terminal fragment comprising amino acids 1 to 157 (N-CirA) and a C-terminal fragment comprising amino acids 158 to 633 (C-CirA) (Fig. 6a). Expression of N-CirA or C-CirA in protein in *ΔcirA* strains (*ΔcirA/*N*-cirA* or *ΔcirA/*C*-cirA*) transformed with the pTV-N-*cirA* or pTV-C-*cirA* strains was observed in bacterial cells and OMVs (Fig. 6b). The increase in expression of iNOS and COX-2 in donor Mφ treated by OMVs was the same in the WT, *ΔcirA* (iNOS, 1.00-fold; COX-2, 0.87-fold), *ΔcirA/*N*-cirA* (iNOS, 0.88-fold; COX-2, 1.18-fold), and *ΔcirA/*C*-cirA* (iNOS, 0.74-fold; COX-2, 1.00-fold) strains (Fig. 6c). In recipient Mφ, expression of *iNos* and *Cox-2* by *ΔcirA/*C-*cirA*-exo (*iNos*, 1.07-fold; *Cox-2*, 2.58-fold) was as same as that by WT-exo, while that by *ΔcirA*-exo (*iNos*, 0.37-fold; *Cox-2*, 0.23-fold) and Δ*cirA*/N-*cirA*-exo (*iNos*, 0.31-fold; *Cox-2*, 0.07-fold) was lower (Fig. 6d). These results show that C-terminal fragment of CirA triggers inflammatory responses via E. coli-derived OMVs and Mφ-derived exosomes.

## DISCUSSION

EVs are membrane-bound vesicles from cells. EVs produced during an infection can be derived from pathogens or the host. Here, we show that E. coli-derived OMVs, and exosomes derived from infected Mφ, rely on inflammatory responses to naive Mφ. Expression of iNOS and COX-2 by naive Mφ increased after exposure to exosomes from live E. coli-infected Mφ, but not Mφ infected by dead (heat-inactivated) E. coli (Fig. 1). As for live E. coli, expression of inflammatory mediators by naive Mφ was increased by exosomes derived from Mφ, which were activated by E. coli-derived OMVs in the absence of bacterial cells (Fig. 2). These data suggest that E. coli-derived OMVs and Mφ-derived exosomes trigger inflammatory responses in Mφ far from the local area of bacterial infection. OMVs, nanostructures ranging from 20 to 300 nm in diameter, are released by almost all Gram-negative bacteria (17, 18). The cargoes include an array of materials derived from the parental bacterium, including outer membrane, periplasmic, and cytosolic proteins, peptidoglycans, LPS, DNA, and RNA (17). Some pathogenic bacteria secrete virulence factors within OMVs, which interact with pathogenically relevant human cells. The OMVs released by enterohemorrhagic E. coli O157 include Siga toxin 2a, cytolethal distending toxin V, and hemolysin (19). OMVs from Enterotoxigenic E. coli also include physiological, active, heat-labile enterotoxin (16). Helicobacter pylori-derived OMVs contain the oncogenic cytotoxin-associated CagA protein, which is a major virulence factor. It is thought that OMVs facilitate direct delivery of virulence factors to target host cells rather than to the external environment, where diffusion has less functional impact (20). So what effect do nonpathogenic bacteria-derived OMVs have on host cells? A previous proteomics analysis of E. coli DH5α-derived OMVs showed that inclusion of particular proteins within OMVs does not appear to be strictly dependent on their abundance, suggesting that specific protein-sorting mechanisms operate during production of OMVs (15). We identified major components of the outer membrane protein, OmpA, and low-abundance outer membrane protein, FepA, in E. coli-derived OMVs. However, it was unclear whether proteins contained in OMVs from nonpathogenic bacteria also have cytotoxic effects on host cells. In contrast, OMV-contained LPS activates cytosolic caspase-11 (12). LPS is an abundant component of OMVs from pro- and nonpathogenic Gram-negative bacteria. Our results show that LPS-treated donor Mφ showed increased expression of inflammatory mediators, while exosomes from LPS-treated Mφ failed to increase expression of these mediators in recipient Mφ (see Fig. S2b in the supplemental material). Therefore, LPS from OMVs does not play a role in exosome-related inflammatory responses. More than 1 ng/mL LPS increased expression of iNOS in naive Mφ (Fig. S2c). However, the LPS concentration in Mφ-derived exosomes isolated from Mφ culture medium was 0.0026 ± 0.0001 ng/mL (Fig. S2d). Thus, LPS in both OMVs and exosomes did not promote the inflammatory responses by exosomes from Mφ that were infected with E. coli. In relation to inflammatory responses relayed by the two EVs, we found that proteins in exosomes were the key factors, while DNA and RNA did not have a role (Fig. 3a and b). The increase in iNOS protein expression mediated by Mφ-derived exosomes was decreased by treatment with proteinase in the presence or absence of Triton X-100. These results show that the key factors that increase expression of iNOS are on the outside and/or inside exosomes. In contrast, the factors inducing COX-2 were presence inside the exosomes because increased expression of COX-2 by Mφ-derived exosomes was decreased by protease with Triton X-100, but not without Triton X-100. We identified the CirA protein as an important factor that relays inflammatory signals. Indeed, the CirA protein was visualized in exosomes from Mφ treated with E. coli-derived OMVs (Fig. 5a). Moreover, exosomes from Mφ treated with OMVs derived from the *ΔcirA* strain caused a slight increase in iNOS expression while strongly suppressing expression of COX-2 (Fig. 4b and Fig. 5e). These results show that proteins other than CirA on the membrane of exosomes must be involved in increased expression of iNOS. CirA, which is a receptor of colicin Ia (21, 22), is expressed in E. coli-derived OMVs (15). CirA is a TonB-dependent transporter; spanning of the periplasmic space is facilitated by the inner membrane protein TonB (22). The transmembrane β-barrel, consisting of residues 164 to 663, contains 22 beta strands connected by 11 long extracellular loops and 10 short periplasmic loops. The N-terminal plug domain interacts with the interior of the β-barrel. The TonB box, comprising residues 26 to 163, resides at the N-terminal end of the plug domain. The TonB box is required for killing of cells by colicin Ia and plays an important role in transport of colicin Ia by CirA. Therefore, we confirmed whether the N-terminal plug domain was required for relay of inflammatory responses from E. coli-derived OMVs to Mφ-derived exosomes. The inflammatory responses were dependent on the C-terminal transmembrane β-barrel, not on the N-terminal plug domain (Fig. 6). Exosomes from Mφ treated with OMVs increased expression of TNF-α, IL-1β, and IL-6 in a CirA-dependent manner (Fig. S6b and Fig. S7). Thus, CirA in Mφ-derived exosomes acts as a proinflammatory factor during infection with E. coli. It is not known whether CirA receptor is present in Mφ. In contrast, OmpA, an outer membrane protein of E. coli, interacts with a 95-kDa glycoprotein on brain microvascular endothelial cells (Egcp), which allows E. coli to breach the blood-brain barrier (23). In the present study, we found that OmpA played no role in exosome-mediated inflammatory responses during infection with E. coli. The OmpA receptor, Egcp, might be not expressed by Mφ. Another outer membrane protein, OmpC, did not play a role in exosome-mediated inflammatory responses. When E. coli lacking OmpC were cultured under different glucose concentrations, expression of CirA on the outer membrane changed (24). Here, we showed that deletion of OmpC did not alter expression of CirA by OMVs (Fig. S3c). Moreover, OmpC was not directly related to exosome-mediated inflammatory responses, but might be a target for inhibiting them. The protein content of OMVs from the *ΔompC* strain was one-tenth that of the WT strain, suggesting that decreased expression of OmpC might suppress release of OMVs (Fig. S4b). Because inflammatory responses were dependent on the dose of OMVs, reducing the amount of OMVs may be one effective way to stop relay of exosome-mediated inflammatory responses from Mφ to Mφ (Fig. S1). Here, we treated donor Mφ with the same amount of protein (10 μg/8 × 10^6^ cells) and found that OMVs from the *ΔompC* strain strongly increased expression of iNOS and COX-2. We considered that high concentrations of E. coli-secreted molecules, including LPS, were contained within OMVs from the *ΔompC* strain. TonB-dependent outer membrane transporters include FecA, the ferric citrate transporter; BtuB, the vitamin B_12_ receptor; FhuA, the ferrochrome receptor; and CirA-FepA, the ferric-enterochelin receptor (25, 26). In our study, we confirmed that the protein components of the TonB-dependent transporter are present in E. coli-derived OMVs. CirA and FepA share the highest sequence similarity (30.8%) within the TonB-dependent group. Moreover, the association between CirA and FepA could be extended to encompass a close evolutionary relationship (25). Both the CirA and FepA transporters play a role in colicine binding receptor and the uptake of ferric iron (Fe^3+^) complexed with siderophores. CirA was identified as a receptor of microcin that is of a lower molecular weight than colicins (27). Thus, the function of CirA and FepA are not exactly the same. On host cells, CirA relays inflammatory responses from donor Mφ to recipient Mφ, while FepA did not. Some studies show that the release of extracellular microvesicles, increased by the lack of iron in a culture system, triggers a stress response in bacterial species such as Haemophilus influenzae, Mycobacterium tuberculosis, and uropathogenic E. coli (28–30). We found that the amount of OMVs released by E. coli also increased as the iron concentration in the culture medium decreased (Fig. S8). We cultured E. coli under iron-limiting conditions (0.2 μM Fe^3+^) to prepare OMVs. Iron depletion increased expression of CirA in E. coli, resulting in the taking up of CirA into OMVs. E. coli K-12 strain-derived OMVs, which were analyzed by Reimer et al., did not contain the TonB-dependent transporters, including CirA (31); this must be because E. coli was cultured in iron-rich LB broth. Indeed, we found that the expression of CirA in bacterial cells decreased in an iron dose-dependent manner when more than 10 μM iron was added to the E. coli culture medium (Fig. S8). This phenomenon was also observed in Salmonella. Thus, CirA appears to be selectively incorporated into OMVs from some CirA-expressing bacteria under iron-limited conditions. Furthermore, a low iron concentration would not only increase in the expression of CirA but also enhance the inflammatory responses relayed by OMVs and exosomes during E. coli infection of the host. Within hours of human infection, the concentration of iron in extracellular fluid and plasma decreases dramatically (32, 33). This is thought to be a critical host defense strategy against bacterial pathogens (34). In contrast, our results suggest that iron-limiting conditions upregulate CirA expression, resulting in relay of inflammatory responses by OMVs and exosomes.

10.1128/mbio.03051-22.7 FIG S7 Complementation of N- and C-terminal *cirA* gene increased the expression of cytokine mRNA by macrophage-derived exosomes. Mouse RAW264.7 cells (recipient cells; 5 × 10^5^ cells) were incubated with exosomes from donor cells (5 × 10^5^ cells) for 24 h. The mRNA expression of tumor necrosis factor alpha (TNF-α), interleukin 1 beta (IL-1β), and IL-6 was analyzed by quantitative real-time PCR and normalized to 18S rRNA. Values are expressed as the mean ± SD (*n* = 4) of at least three independent biological replicates. One-way ANOVA and Tukey’s test were used for statistical analysis; *, *P* < 0.05; **, *P* < 0.01; ***, *P* < 0.005; ****, *P* < 0.001. Download FIG S7, PDF file, 0.1 MB. Copyright © 2023 Imamiya et al. 2023 Imamiya et al. https://creativecommons.org/licenses/by/4.0/ This content is distributed under the terms of the Creative Commons Attribution 4.0 International license.

10.1128/mbio.03051-22.8 FIG S8 Iron concentration and CirA expression. The E. coli K-12 strain and Salmonella enterica serovar Enteritidis IID604 strain were cultured with DMEM containing 0 to 100 μM FeCl_3_ at 37°C for 12 h. Bacteria lysates were analyzed by Western blotting. Download FIG S8, PDF file, 0.3 MB. Copyright © 2023 Imamiya et al. 2023 Imamiya et al. https://creativecommons.org/licenses/by/4.0/ This content is distributed under the terms of the Creative Commons Attribution 4.0 International license.

In the present study, we identified Mφ-derived exosomes by measuring particle size and confirming marker proteins. However, it must contain other extracellular vesicles of different origin than the exosome. Budden et al. (35) characterized the extracellular vesicle released by human macrophage THP-1 cells upon activation of the NLRP3 inflammasome. Therefore, we examined whether E. coli-derived OMVs induced NLRP3 activation, resulting in activation of caspase-1 and pyroptosis via processing gasdermin D. Caspase-1 was activated in mouse BMDM after stimulation by E. coli-derived OMVs (Fig. S9f). In contrast, cleaved caspase-1 was not detected in RAW264.7 cells because ASC, a factor in the inflammasome complex, was reported to be completely absent in RAW264.7 cells (Fig. S9a). Furthermore, E. coli-derived OMVs are known to be sensed by caspase-11 and to activate the cell death-inducing protein gasdermin D independently of caspase-1 (12). In fact, the expression of caspase-11 was stimulated in both RAW264.7 cells and mouse BMDM; however, gasdermin D was activated only in mouse BMDM (Fig. S9b, c, f, and g). Intracellular ATP levels were decreased by E. coli-derived OMVs, while cell proliferation measured by 3-(4,5-dimethyl-2-thiazolyl)-2,5-diphenyl-2H-tetrazolium bromide (MTT) assay resulted in an increase by E. coli-derived OMVs (Fig. S9d, e, i, and j). In addition, the extracellular lactate dehydrogenase, as cell death, was not detected in culture medium by Mφ treated with or without E. coli-derived OMVs (data not shown). These data suggest that the dose of E. coli-derived OMVs used in this study caused little pyroptosis. In fact, more than 50 μg of OMVs was reported to cause pyroptosis (12).

10.1128/mbio.03051-22.9 FIG S9 Activation of inflammasome and cell death in OMV-treated macrophages. Mouse RAW264.7 macrophages (Mφ) (a to e) and mouse bone marrow-derived Mφ (f to j) were incubated with each E. coli-derived OMV (10 μg/7 mL) for 9 h. After culture medium was replaced with fresh medium containing antibiotics, cells were incubated for 1 h. After replacement with fresh media containing antibiotics again, cells were incubated for 9 h. (a, f) The protein expressions of caspase-1 and cleaved caspase-1 in whole-cell lysates were measured by Western blotting. (b, g) The protein expression of caspase-11 and cleaved caspase-11 in whole-cell lysates was measured by Western blotting. (c, h) The protein expression of gasdermin D and cleaved gasdermin D in whole-cell lysates was measured by Western blotting. β-Actin was used as loading control. (d, i) Luciferase activity was measured as the amount of ATP by using FilterMax F5 multimode microplate reader. (e, j) Cell viability was analyzed by WST assay. Absorbance (Abs) was measured at 450 nm as the number of viable cells by using FilterMax F5 multimode microplate reader. A *t* test was used for statistical analysis. *, *P* < 0.05. Download FIG S9, TIF file, 0.6 MB. Copyright © 2023 Imamiya et al. 2023 Imamiya et al. https://creativecommons.org/licenses/by/4.0/ This content is distributed under the terms of the Creative Commons Attribution 4.0 International license.

We demonstrate that the CirA protein in the OMVs from live E. coli is incorporated into Mφ-derived exosomes and increases protein expression of inflammatory mediators. However, proteins other than CirA may play roles in exosome-mediated inflammatory responses. In particular, increased expression of iNOS was partially attenuated by the *ΔcirA* E. coli strain. Other factors will be clarified in a future study.

## MATERIALS AND METHODS

### Materials.

RAW264.2 cells were purchased from ATCC. All plasmids and bacterial strains used in this study are shown in Tables 2 to 4. E. coli K-12 BW25113 (wild type [WT]) and single gene-deleted mutant strains (*ΔompA*: *opmA*::*kan* for JW0940-KC, *ΔfepA*: *fepA*::*kan* for JW5086-KC, *ΔcirA*: *cirA*::*kan* for JW2142-KC, *ΔdegP*: *degP*::*kan* for JW0157-KC, and *ΔompC*: *ompC*::*kan* for JW2203-KC) were provided by National BioResource Project (NIG, Japan). The competent cell BL21(DE3) strain and DH5α strain were purchased from TaKaRa (Shiga, Japan). LB broth was purchased from Sigma/Merck (Tokyo, Japan). The plasmid pET22b(+) was purchased from Novagen/Merck (Tokyo, Japan). pTV118N vector was gifted by Ishijima (Kyoto Prefectural University). Ex Taq, Mighty TA-cloning kit containing pMD T vector, DNA ligation kit Mighty Mix, in-fusion HD cloning kit, isopropyl β-d-1-thiogalactopyranoside (IPTG), and TB Green premix Ex Taq II were purchased from TaKaRa (Shiga, Japan). Ni-nitrilotriacetic acid (NTA) agarose was purchased from Qiagen (Hilden, Germany). cOmplete Mini protease inhibitor was purchased from Roche/Nippon Gene (Tokyo, Japan). Anti-OmpC antibody was purchased from MyBioSource (Vancouver, Canada). Isogen II was purchased from Nippon Gene (Tokyo, Japan). Can Get Signal and ReverTra Ace quantitative PCR (qPCR) reverse transcription (RT) kit were purchased from Toyobo (Osaka, Japan).

**TABLE 2:** Bacteria strains used in this study^*a*^

E. coli strain	Gene type	Source
WT	Wild type	K-12 BW25113
*ΔompA*	*ompA* deletion::*kan*	JW0940-KC
*ΔfepA*	*fepA* deletion::*kan*	JW5086-KC
*ΔcirA*	*cirA* deletion::*kan*	JW2142-KC
*ΔcirA/pTV*	*cirA* deletion::*kan* transfected pTV118N vector	This study
*ΔcirA/cirA*	*cirA* deletion::*kan*::*cirA* derivative carrying pTV-*cirA* vector	This study
*ΔcirA/NcirA*	*cirA* deletion::*kan*::N-terminal *cirA* derivative carrying pTV-N*cirA* vector	This study
*ΔcirA/CcirA*	*cirA* deletion::*kan*::C-terminal *cirA* derivative carrying pTV-C*cirA* vector	This study
*ΔdepP*	*degP* deletion::*kan*	JW0157-KC
*ΔompC*	*ompC* deletion::*kan*	JW2203-KC

a E. coli K-12 BW25113 (wild type [WT]) and single gene-deleted mutant strains (*ΔompA*: *opmA*::*kan* for JW0940-KC, *ΔfepA*: *fepA*::*kan* for JW5086-KC, *ΔcirA*: *cirA*::*kan* for JW2142-KC, *ΔdegP*: *degP*::*kan* for JW0157-KC, and *ΔompC*: *ompC*::*kan* for JW2203-KC) were provided by the National BioResource Project (NIG, Japan). Each gene-complementary strain, *ΔcirA/cirA*, *ΔcirA*/N*cirA*, and *ΔcirA*/C*cirA*, was the transformation of pTV-*cirA*, pTV-N*cirA*, and pTV-C*cirA* into the Δ*cirA* strain, respectively. The *ΔcirA/pTV* strain was the transformation of pTV118N empty vector as the control.

**TABLE 3:** Primers used in this study^*a*^

Name	Sequence (5′–3′)	Template	For construction of:
pMD-cirANdeI-s	CCCCATATGTTTAGGTTGAACCCTTTCGTACGG	E. coli genome DNA	pMD-cirA
pMD-cirANotI-a	CCCGCGGCCGCGAAGCGATAATCCACTGCC	E. coli genome DNA	pMD-cirA
pTV-cirANcoI-s	**GTAATCATGGCCATG**TCAGTGGTGGTGGTGGTG	pET-cirA	pTV-cirA
pTVcirANcoI-a	**AGGAAACAGAC**CATGATGTTTAGGTTGAACCCTTTC	pET-cirA	pTV-cirA
pET-NcirANcoI-s	**CAGCCGGCGATGGCCATGG**CCATGTTTAGGTTG	pET-cirA	pET-NcirA
pET-NcirAXhoI-a	**GTGGTGCTCGAGTGC**GGTGATGATATTC	pET-cirA	pET-NcirA
pTV-NcirANcoI-s	**GTAATCATGGCCATG**TCAGTGGTGGTGGTGGTG	pET-NcirA	pTV-NcirA
pTV-NcirANcoI-a	**AGGAAACAGAC**CATGTTTAGGTTGAACCC	pET-NcirA	pTV-NcirA
pET-CcirANcoI-s	**CAGCCGGCGATGGCCATG**AAAAAAATCGGTC	pET-cirA	pET-CcirA
pET-CcirAXhoI-a	**GTGGTGCTCGAGTGC**GAAGCGATAATCC	pET-cirA	pET-CcirA
pTV-CcirANcoI-s	**AGGAAACAGACCATG**AAAAAAATCGG	pET-CcirA	pTV-CcirA
pTV-CcirANcoI-a	**AGGAAACAGAC**CATGATGTTTAGGTTGAACCCTTTC	pET-CcirA	pTV-CcirA
pMD-degPNdeI-s	CCCCATATGAAAAAAATTAGCACTGAGTGC	E. coli genome DNA	pMD-degP
pMD-degPHindIII-a	CCCAAGCTTCTGCATTAACAGGTAGATGGTGC	E. coli genome DNA	pMD-degP
pET-ompANdeI-s	**AAGGAGATATACAT**ATGAAAAAGACAGCTATCGCG	E. coli genome DNA	pET-ompA
pET-ompAXhoI-a	**GGTGGTGGTGCTCGAG**AGCCTGCGGCTGAGTTAC	E. coli genome DNA	pET-ompA
pET-fepANdeI-s	**AAGGAGATATACAT**ATGAACAAGAAGATTCATTCC	E. coli genome DNA	pET-fepA
pET-fepAXhoI-a	**GGTGGTGGTGCTCGAG**GAAGTGGGTGTTTACGC	E. coli genome DNA	pET-fepA

a The gene amplified using primers of forward (s) and reverse (a) was cloned into each vector. Single underlines represent sequences as restriction enzyme. Bold represent reverse complementary sequences for the In-Fusion cloning.

**TABLE 4:** List of plasmids used for real-time PCR

Gene name	Accession no.	Forward (5′–3′) sequence	Reverse (5′–3′) sequence
18S rRNA	NR_003278	GCAATTATTCCCCATGAACG	AGGGCCTCACTAAACCATCC
*iNos*	NM_010927	GTTCTCAGCCCAACAATACAAGA	GTGGACGGGTCGATGTCAC
*Cox-2*	NM_011198	GATGCTCTTCCGAGCTGTG	GGATTGGAACAGCAAGGATTT
TNF-α	NM_013693	AAGCCTGTAGCCCACGTCGTA	GGCACCACTAGTTGGTTGTCTTTG
IL-1β	NM_008361	CCCAAGCAATACCCAAAGAA	CATCAGAGGCAAGGAGGAAA
IL-6	NM_031168	CCATCCAGTTGCCTTCTTG	AAGTGCATCATCGTTGTTCATAC

### Plasmids and bacterial strains.

Cloning of the E. coli *cirA* gene (GenPept accession no. NP_416660) was amplified using Ex Taq with the primers pMD-*cirA*NdeI-s and pMD*cirA*NotI-a and E. coli (WT) genome DNA as the template. The pMD-*cirA* plasmid was ligated with the amplified fragments and pMD T vector by using Ligation Mighty Mix (TaKaRa). The pET-*cirA* plasmid was ligated with the fragments *cirA*NdeI-NotI of pMD-*cirA* digested by NdeI and NotI restriction enzymes and the NdeI-NotI-digested pET-22b(+) plasmid by using ligation mix (TaKaRa). The cirA-6×His fragments were amplified with the primers pTV-*cirA*NcoI-s and pTV-*cirA*NcoI-a and pET-*cirA* as the template. The pTV-*cirA* plasmid was ligated with the amplified fragment of cirA-6×His and NcoI-digested pTV plasmid by the In-Fusion HD. The fragment of N/C-*cirA* was amplified with plasmids pET-N*cirA*NcoI-s and pET-N*cirA*NotI-a, or pET-C*cirA*NcoI-s and pET-C*cirA*NotI-a, and pET-*cirA* plasmid as the template. The pET-N/C*cirA* plasmids were ligated with amplified fragments N/C*cirA*, and NcoI-digested pET plasmid by In-Fusion HD. The N/C*cirA*-6×His fragments were amplified with the primers pTV-N/C*cirA*NcoI-s and pTV-N/C*cirA*NcoI-a and pET-N/C*cirA* as the template. The pTV-N/C*cirA* plasmids were ligated with the amplified fragment of N/C*cirA*-6×His and NcoI-digested pTV plasmid by In-Fusion HD. Cloning of the E. coli *degP* gene (GenPept accession no. NP_414703) was amplified with the primers pMD-*degP*NdeI-s and pMD-*degP*HindIII-a and E. coli (WT) genome DNA as the template. The pMD-*degP* plasmid was ligated with the amplified fragments and pMD T vector by using Ligation Mighty Mix. The pET-*degP* plasmid was ligated with the fragments *degP*NdeI-HindIII of pMD-*degP* digested by NdeI and HindIII restriction enzymes and the NdeI-HindIII-digested pET-22b(+) plasmid by using ligation mix. Cloning of the E. coli *ompA* gene (GenPept accession no. NP_415477) and *fepA* gene (GenPept accession no. NP_415116) were amplified with the primers pMD-*ompA*NdeI-s and pMD-*ompA*XhoI-a, or pMD-*fepA*NdeI-s and pMD-*fepA*XhoI-a, and E. coli (WT) genome DNA as the template. The plasmid of pET-*ompA* or pET-*fepA* was ligated with the amplified fragment of *ompA* or *fepA* and NcoI-digested pET plasmid by In-Fusion HD. Each gene-containing coding region was designed to allow the expression of a C-terminal, 6× His-tagged variant of recombinant proteins following ligation into the pET-22b(+) plasmid. After construction, the integrity of some expression vectors was confirmed by DNA sequencing. The gene-complementary strains, Δ*cirA/cirA*, Δ*cirA*/*NcirA*, and Δ*cirA*/*CcirA*, were obtained through the transformation of pTV-*cirA*, pTV-*NcirA*, and pTV-*CcirA* into Δ*cirA* strain using electroporation (240 KV, 13 F, 243 Ω; Cell Porator; Gibco). These strains were cultured in LB broth with 25 μg/mL for kanamycin and 100 μg/mL for ampicillin (Wako).

### Recombinant protein.

The expression vectors (pET-*ompA*, pET-*fepA*, pET-*cirA*, and pET-*degP*) were transformed into the competent E. coli strain BL21(DE3) strain. Bacteria were incubated in LB broth with 100 μg/mL ampicillin at 37°C with shaking until the optical density at 600 nm (OD_600_) reached about 0.6. After incubation of bacteria at 22°C overnight with 0.1 mM IPTG, the bacterial cells were pelleted at 2,000 × *g* at 4°C for 30 min, and the supernatant was removed. The cells were lysed by lysis buffer (20 mM Tris-HCl, pH 8.0, 500 mM NaCl, 10% [vol/vol] glycerol, and 8 M urea) and were completely disrupted by sonication. After the centrifugation at 2,000 × *g* at 4°C for 30 min, the supernatant was filtrated through a 0.45-μm filter (Kurabo, Osaka, Japan). The filtrate was loaded onto an Ni-NTA agarose column. The column was washed with binding buffer (10 mM imidazole, 50 mM NaH_2_PO_4_, 500 mM NaCl, and 0.01% [vol/vol] Tween 20, pH 8.0) and wash buffer (30 mM imidazole, 50 mM NaH_2_PO_4_, 500 mM NaCl, and 0.01% [vol/vol] Tween 20, pH 8.0). The recombinant protein was eluted with elution buffer (300 mM imidazole, 50 mM NaH_2_PO_4_, 500 mM NaCl, and 0.01% [vol/vol] Tween 20, pH 8.0).

### Antibody preparation.

Four-week-old female BALB/cCrSlc mice (SLC, Shizuoka, Japan) were subcutaneously injected with 10 μg of each recombinant protein (OmpA, FepA, CirA, or DegP) mixed with Freund’s incomplete adjuvant (BD, Tokyo, Japan) twice at 2-week intervals. Mouse serum 21 days after the second immunization was used to analyze each recombinant protein or E. coli lysate by Western blotting.

### E. coli culture and preparation of Ec-med, Ec-sup, and OMVs.

The E. coli DH5α strain, E. coli K-12 BW25113 strain (WT), and E. coli deletion mutants were cultured at 37°C in LB broth. Bacteria were prepared at an OD_600_ of 0.0008 by Dulbecco's modified Eagle medium (DMEM) containing 4.5 g/L glucose and 0.2 μM Fe^3+^ (Fujifilm, Osaka, Japan) and then were cultured at 37°C for 12 h under 5% CO_2_ conditions. ClearColi (E. coli BL21(DE3) mutant lacking the oligosaccharide chain of LPS; Lucigen, Buenos Aires, Argentina) was gifted from Sohkichi Matsumoto (Department of Bacteriology, Niigata University Graduate School of Medical and Dental Sciences). Similarly, ClearColi bacteria were cultured at 37°C for 27 h under 5% CO_2_ conditions. After centrifugation at 2,000 × *g* for 30 min, the bacterial culture medium was collected as supernatant. After we centrifuged the bacterial culture medium at 10,000 × *g* at 4°C for 30 min, the supernatant was filtrated by a 0.2-μm filter (Kurabo). After we ultracentrifuged the filtrate (Ec-med) using a 50.2 Ti rotor (Beckman) at 100,000 × *g* at 4°C for 3 h, the supernatant (Ec-sup) was removed, and phosphate-buffered saline (PBS) was added in pellets. After we ultracentrifuged the filtrate at 100,000 × *g* at 4°C for 2 h, pellets (OMVs) were suspended by PBS.

### Density gradient of OMVs.

After we ultracentrifuged the filtrate at 100,000 × *g* at 4°C for 3 h, pellets suspended by PBS were used as OMVs. Moreover, the OMVs were purified using an OptiPrep (Axis-Shield Diagnostics, XA, Scotland) density gradient. A discontinuous iodixanol gradient was prepared by diluting a stock solution of OptiPrep (60%, wt/vol) with 10 mM Tris, pH 7.5, containing 0.25 M sucrose to generate 40%, 20%, 10%, and 5% (wt/vol) iodixanol solutions. The discontinuous iodixanol gradient was generated by sequentially layering 3.0 mL of 40% (wt/vol) iodixanol solutions, 3.0 mL of 20% (wt/vol) iodixanol solutions, 3.0 mL of 10% (wt/vol) iodixanol solutions, and 2.5 mL of 5% (wt/vol) iodixanol solutions in a centrifuge 344060 tube (Beckman). A 0.3-mL volume of OMVs in PBS was overlaid on discontinuous iodixanol gradient and ultracentrifuged using an SW40 Ti rotor (Beckman) at 160,000 × *g* at 4°C for 16 h. Twelve fractions of 1 mL each were collected from the top of the iodixanol solution and used for analysis such as Western blotting.

### TEM analysis.

OMV samples were applied to the carbon film TEM grids, which were created by carbon evaporation using VE-2020 (Vacuum Device, Tokyo, Japan). After staining with 1% uranyl acetate, samples were observed in a Talos F200C G2 (Thermo).

### Donor cell culture.

RAW264.7 cells (8 × 10^6^ cells) were cultured in 7 mL of DMEM containing 10% fetal bovine serum (Biowest, Tokyo, Japan) and 100 units/mL penicillin and 100 μg/mL streptomycin (Gibco/Thermo, Tokyo, Japan). After preincubation for 24 h, the medium was changed to fresh DMEM without antibiotics. Bone marrow was flushed from the femur and tibia of C57BL/6 mice (male, 5 weeks old), and cells were plated in dishes with DMEM containing 10% fetal bovine serum (FBS), 4.5 g/L glucose, and 20% conditioned medium from the supernatants of L929 (LC14) fibroblasts secreting macrophage colony-stimulating factor. Bone marrow cells were differentiated into macrophages in 7 to 10 days and were then used in experiments (36). All mice were maintained under specific-pathogen-free conditions in the animal facilities of Kyoto Prefectural University. After live or heat-inactivated E. coli DH5α and E. coli BW25113 were added to cells at an MOI of 5 for 9 h, culture media were replaced with fresh DMEM medium containing antibiotics, and the cells were incubated for 1 h. After the medium was replaced again with fresh DMEM medium containing antibiotics, cells were incubated for 9 h. After Ec-med, Ec-sup, or OMVs were added to cells for 9 h, culture media were replaced with fresh DMEM medium containing antibiotics, and the cells were incubated for 1 h. After the medium was replaced again with fresh DMEM medium containing antibiotics, cells were incubated for 9 h. The culture medium was collected for preparation of exosomes, and cells were used for analyzing immunoblotting or real-time PCR.

### ATP measurement.

RAW264.7 cells and mouse bone marrow-derived macrophages (BMDMs) (3 × 10^3^ cells/well) were spread in a 96-well plate and cultured for 24 h at 37°C. After E. coli-derived OMVs (0.143 μg/0.1 mL/well) were added to cells for 9 h, culture media were replaced with fresh DMEM medium containing antibiotics, and the cells were incubated for 1 h. After the medium was replaced again with fresh DMEM medium containing antibiotics, cells were incubated for 9 h. After the addition of CellTiterGlo (Promega, Madison, WI) into each well, luciferase activity was measured as the amount of ATP by using a FilterMax F5 multimode microplate reader (Molecular Devices, Osaka, Japan).

### Water-soluble tetrazolium salt (WST) assay.

RAW264.7 cells and mouse BMDMs (5 × 10^3^ cells/well) were spread in a 96-well plate and cultured for 24 h at 37°C. After E. coli-derived OMVs (0.143 μg/0.1 mL/well) were added to cells for 9 h, culture media were replaced with fresh DMEM medium containing antibiotics, and the cells were incubated for 1 h. After replacement again with fresh DMEM medium containing antibiotics, cells were incubated for 9 h. After addition of cell counting kit-8 solution (Dojindo, Kumamoto, Japan) into each well, absorbance (Abs) was measured for 4 h at 450 nm as the number of viable cells by using a FilterMax F5 multimode microplate reader (Molecular Devices, Osaka, Japan). The ratio was calculated as [Abs_(2h)_ − Abs_(1h)_ of OMVs-treated cells]/[Abs_(2h)_ − Abs_(1h)_ of untreated cells] from absorbance of change for 1 h.

### Exosome preparation.

The culture medium from donor cells was centrifuged at 2,000 × *g* at 4°C for 30 min and at 10,000 × *g* at 4°C for 30 min to remove the debris. The supernatant was filtrated through a 0.2-μm filter. After the filtrate was ultracentrifuged using a 50.2 Ti rotor at 100,000 × *g* at 4°C for 3 h, the supernatant was removed. The pellet was ultracentrifuged at 100,000 × *g* at 4°C for 2 h with PBS. The final pellet was suspended in PBS as exosome. The exosomes (0.2 μg) from Mφ, which were infected with live E. coli for 18 h, were incubated with or without 0.01% Triton X-100 at room temperature for 15 min. After incubating the samples with 150 μg/mL proteinase K (Thermo), 0.3 U/mL DNase, or 100 μg/mL RNase at 37°C for 30 min, these were added to the cells (1 × 10^5^ cells/0.3 mL).

### Particle size analysis.

The exosomes prepared by ultracentrifugation were analyzed by using a NanoSight (Quantum Design, Tokyo, Japan) to establish the concentration, size, and intensity. PBS used for dilutions was also measured by using NanoSight to further ensure that there was no contamination. Once the desired concentration of exosomes was reached, the sample was injected into the sample chamber of the NanoSight, and the particle size distribution was obtained through nanoparticle tracking analysis.

### LPS concentrations.

The concentration of LPS in exosomes prepared by ultracentrifugation was analyzed by *Limulus* amebocyte lysate (LAL) assay (Quantum Design, Tokyo, Japan).

### Recipient cell cultures.

RAW264.7 cells were cultured by DMEM containing 10% FBS and antibiotics. After preincubation for 24 h, the medium was changed to fresh maintenance medium, and exosomes were added. For each experiment, the concentrations of exosomes in recipient cells are shown in figure legends. After incubation for 12 h, the cells were analyzed for the expression of mRNA by real-time PCR. After incubation for 24 h, the cells were analyzed for the expression of proteins by immunoblotting.

### Western blotting.

The cells were lysed by BugBuster (EMD/Merck, Darmstadt, Germany) or radioimmunoprecipitation assay (RIPA) buffer (10 mM Tris-HCl, pH 7.4, 5 mM EDTA, pH 8.0, 3.5 mM sodium dodecyl sulfate [SDS], 1% Triton X-100, 24 mM sodium deoxycholate, 199 nM Nα-tosyl-l-phenylalanine chloromethyl ketone, 100 nM tosyl-l-lysyl-chloromethane hydrochloride, 150 mM NaCl, 0.05 M NaF, 25 mM β-glycerophosphate pentahydrate, 1 mM Na_3_VO_4_, and 1 tablet/10 mL of cOmplete Mini). The cell lysate was centrifuged at 5,000 × *g* for 5 min at 4°C, and the supernatants were used to analyze the expression of proteins by Western blotting. The bacteria were lysed by lysis buffer (20 mM Tris-HCl, pH 8.0, 500 mM NaCl, 10%[vol/vol] glycerol, and 8 M urea) and were completely disrupted by sonication. After centrifugation at 2,000 × *g* at 4°C for 30 min, the supernatants were used to analyze the expression of proteins by Western blotting. The samples were suspended in SDS buffer (30 mM Tris-HCl, pH 8.8, 1% SDS, 1% 2-mercaptoethanol, 3.67% glycerol, and 0.017% bromophenol blue) and heated at 95°C for 5 min. Each sample was applied on 7.5 to 15% polyacrylamide gels and immunoblotted against iNOS (1:1,000; BD), COX-2 (1:1,000; Cell Signaling), β-actin (1:5,000; Sigma), integrin β1 (1:1,000; Santa Cruz), HSP90 (1:1,000; BD), CD63 (1:1,000; Santa Cruz), GAPDH (1:1,000; Cell Signaling), CD9 (1:1,000; Abcam), caspase-1 (1:1,000; Cell Signaling), cleaved caspase-1 (1:1,000; Cell Signaling), caspase-11 (1:1,000; Novus), gasdermin D (1:1,000; Cell Signaling), 6×His (1:1,000; Bethyl Laboratories), OmpA (1:1,000), CirA (1:1,000), DegP (1:1,000), FepA (1:1,000), and OmpC (1:1,000) diluted in Can Get Signal or 0.5% skim milk. Blotted proteins were visualized with horseradish peroxidase (HRP)-conjugated goat anti-rabbit IgG or goat anti-mouse IgG antibody and Immobilon Western chemiluminescent HRP substrate. The amount of protein detected by some antibodies was measured using a computed image analysis system (LuminoGraph I; Atto, Osaka, Japan). ImageJ and CS Analyzer 3.0 (Atto) were used for image analysis.

### Isolation of total RNA and real-time PCR.

Total RNA (0.5 μg), extracted from cells by using Isogen II according to the manufacturer’s instructions, was transcribed into cDNA with the ReverTra Ace qPCR RT master mix with a genomic DNA (gDNA) remover in a total volume of 10 μL according to the manufacturer’s instructions. cDNAs were then used as the templates for PCR amplification using TB Green premix Ex Taq II with LightCycler 96 (Roche/Nippon Gene, Tokyo, Japan). Interpolated values for each sample were divided by the corresponding values for 18S rRNA as the housekeeping gene, and the results obtained were expressed as fold changes for a specific gene/18S rRNA using a threshold cycle (ΔΔ*CT*) analysis. Real-time PCR was performed at 95°C for 5 min, followed by 45 cycles at 95°C for 10 s, 60°C for 10 s, and 72°C for 10 s. Primers are shown in Table 4.

### Statistical analysis.

Experiments were performed three times or more. All data are presented as the means ± standard deviations (SDs) of at least three independent biological replicates. Comparisons among groups were performed using a one-way analysis of variance (ANOVA) followed by Tukey’s test or a two-way ANOVA followed by the Bonferroni test. Statistical analyses were performed with GraphPad Prism (Version 9.0). Differences were significant at a *P* value of <0.05.
