# Supplementary figures and images for: Escherichia coli-Derived Outer Membrane Vesicles Relay Inflammatory Responses to Macrophage-Derived Exosomes
Source: mBio. 2023 Jan 17;14(1):e03051-22. doi: 10.1128/mbio.03051-22 (PMC9973271; doi:10.1128/mbio.03051-22)

**a**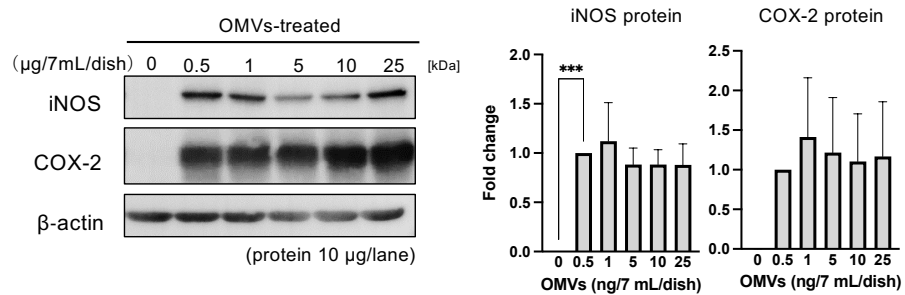**b**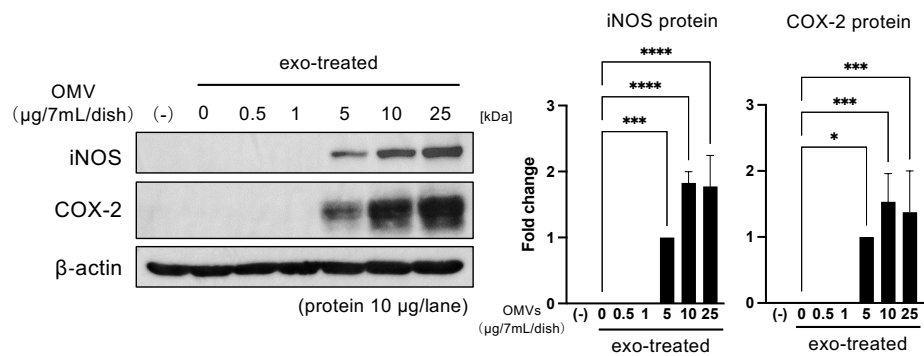

Supplement: FIG S1 [file mbio.03051-22-s0001.pdf]

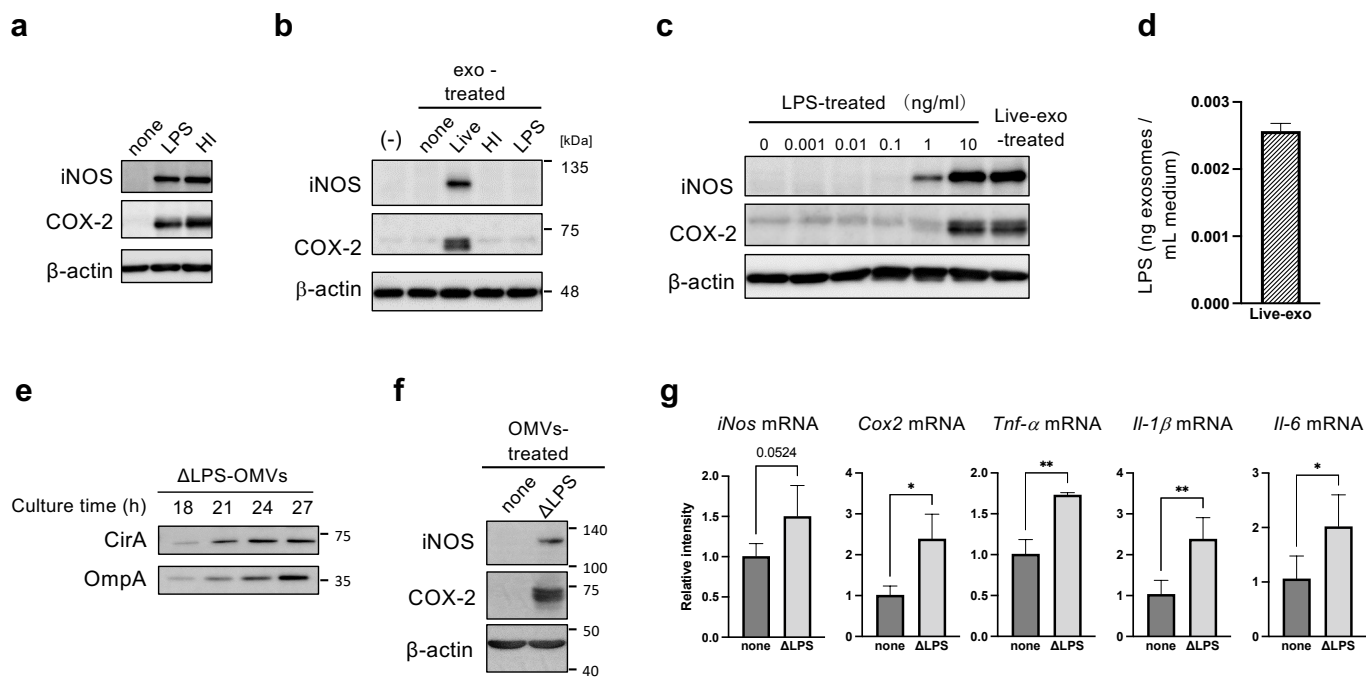

Supplement: FIG S2 [file mbio.03051-22-s0002.pdf]

**a**

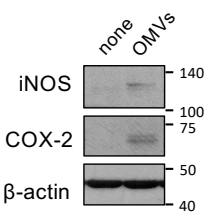

**b**

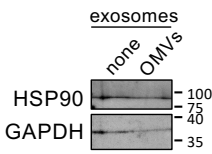

**c**

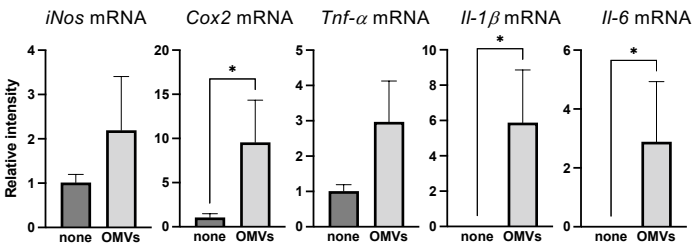

Supplement: FIG S3 [file mbio.03051-22-s0003.pdf]

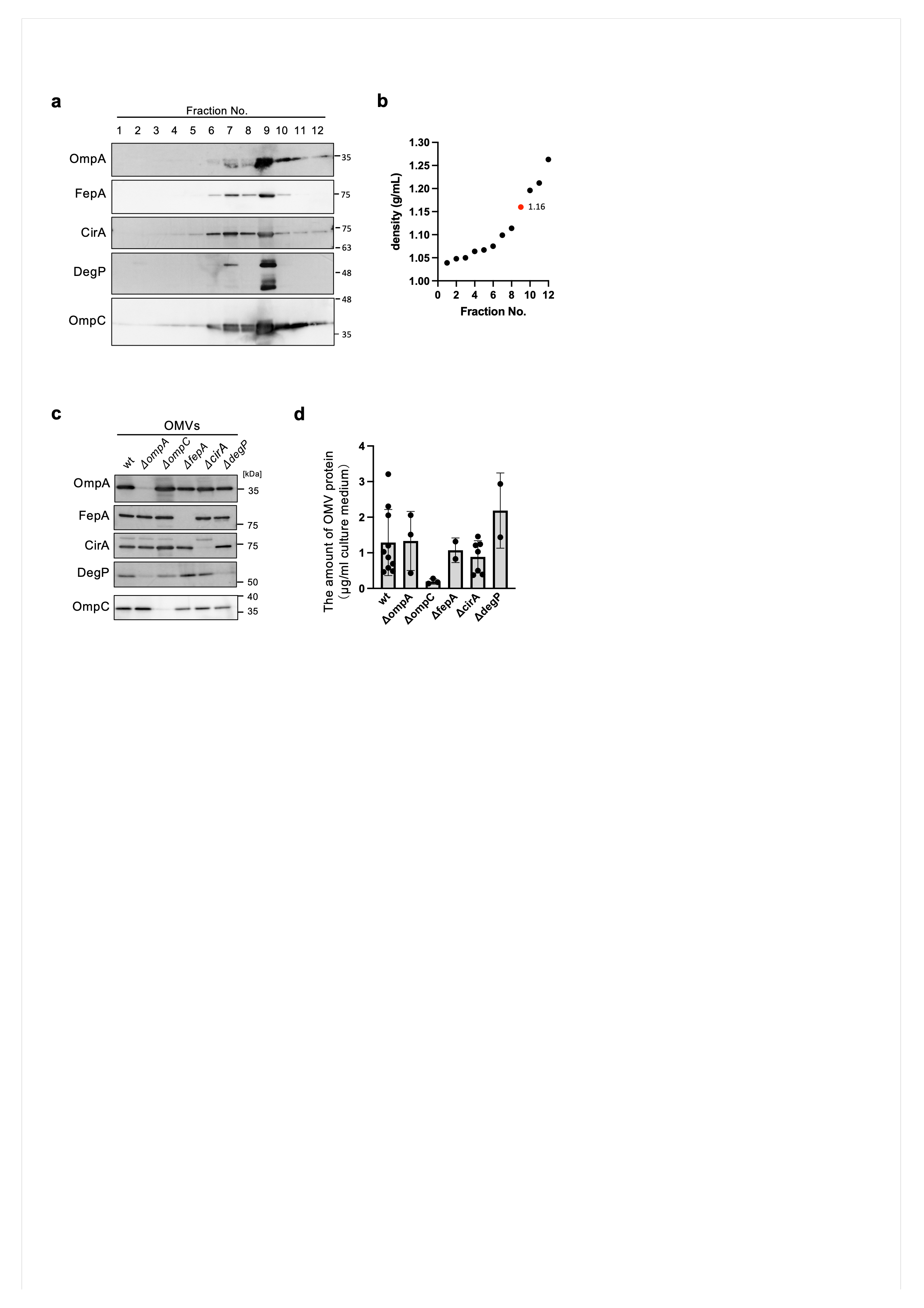

Supplement: FIG S4 [file mbio.03051-22-s0004.tif]

a

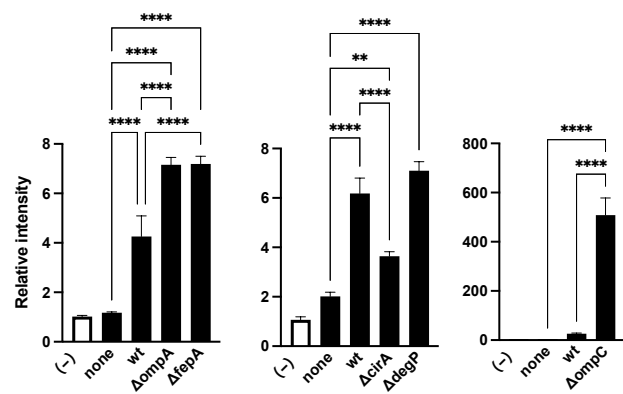

b

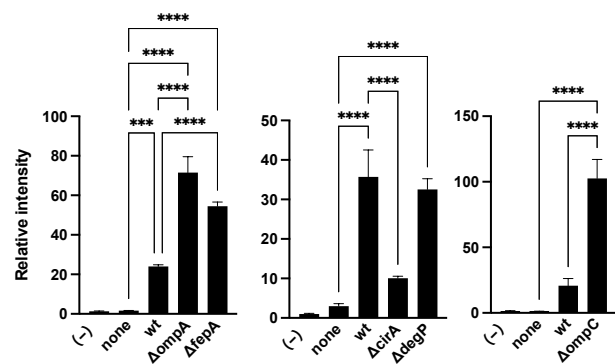

c

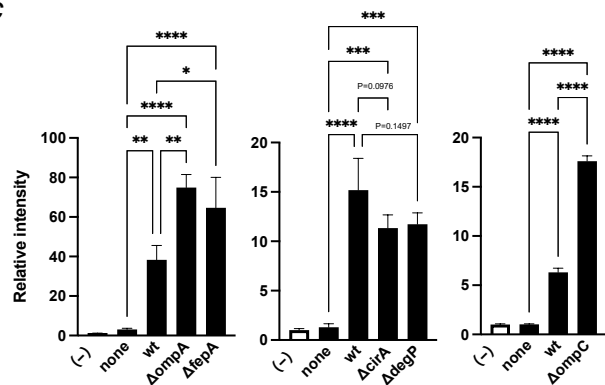

Supplement: FIG S5 [file mbio.03051-22-s0005.pdf]

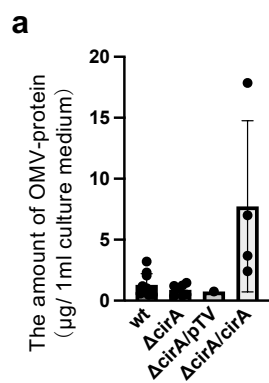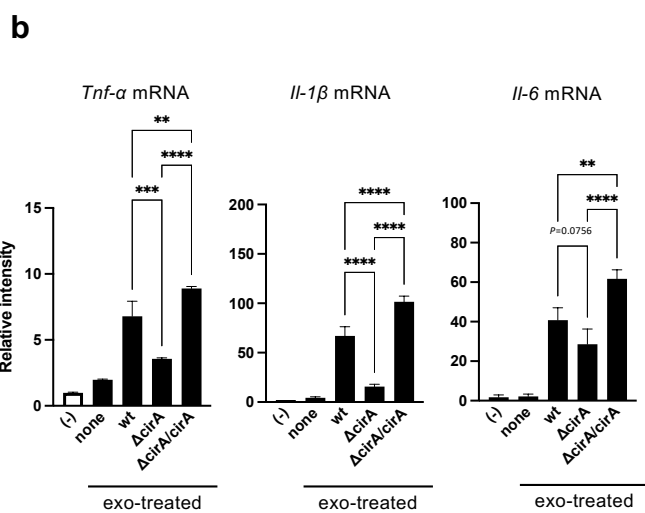

Supplement: FIG S6 [file mbio.03051-22-s0006.pdf]

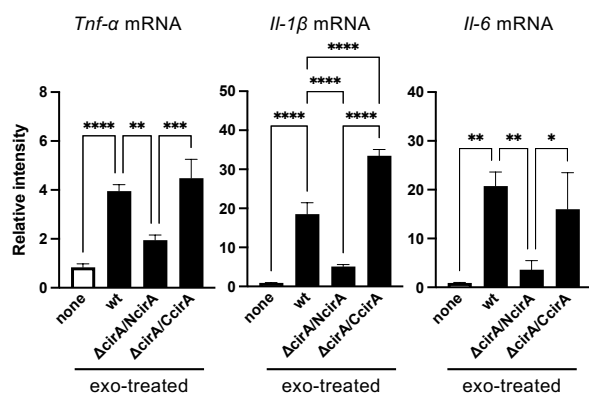

Supplement: FIG S7 [file mbio.03051-22-s0007.pdf]

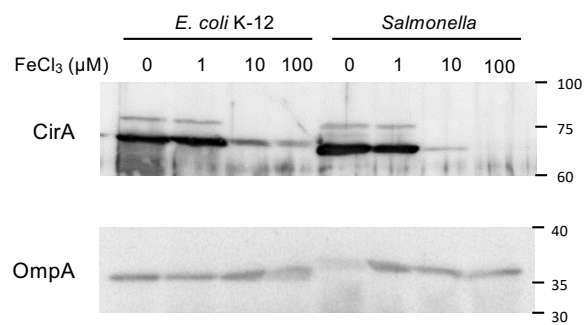

Supplement: FIG S8 [file mbio.03051-22-s0008.pdf]

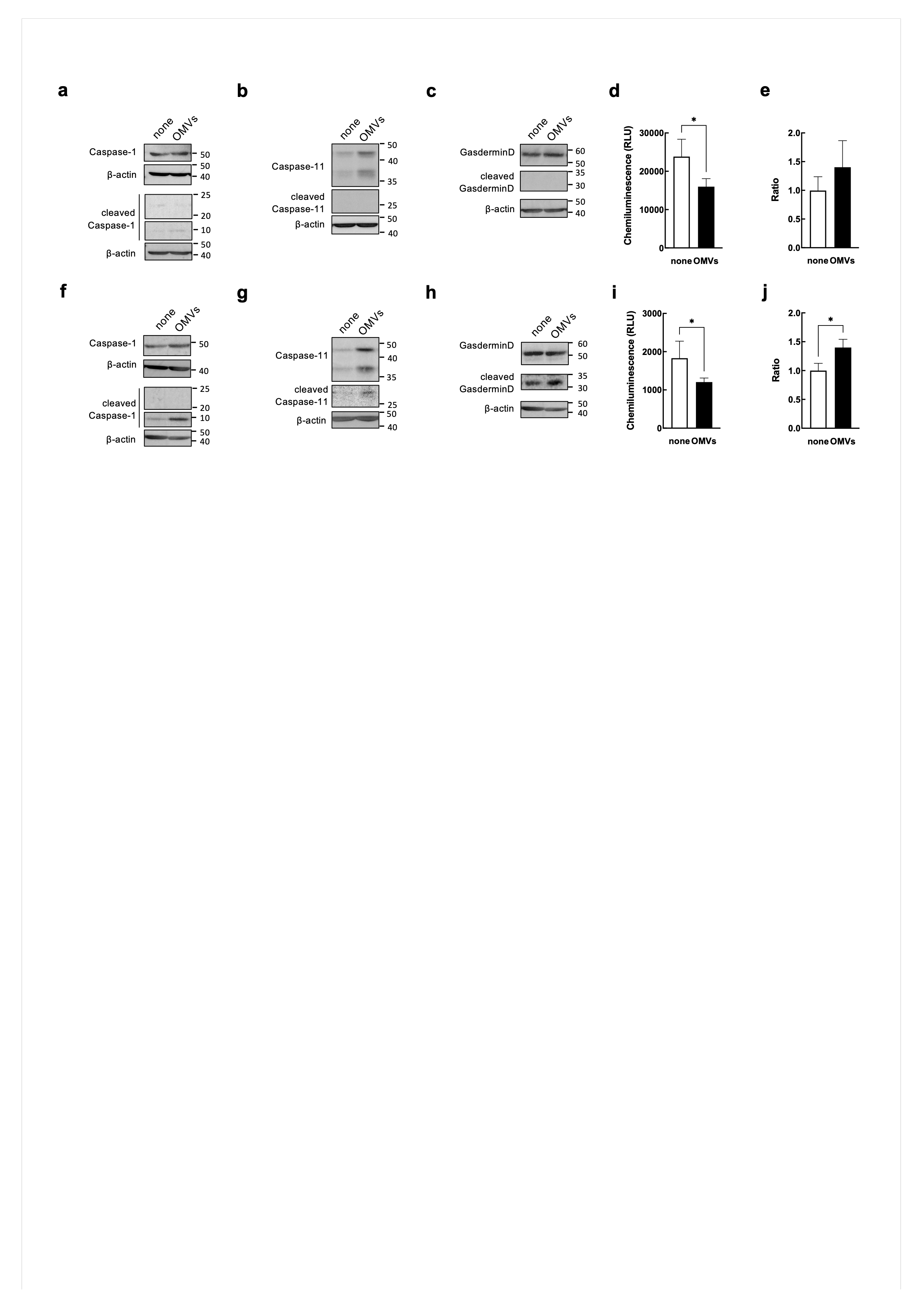

Supplement: FIG S9 [file mbio.03051-22-s0009.tif]
